# Charge-Carrier Behavior and Molecular Interface Engineering in Perovskite Light-Emitting Diodes

**DOI:** 10.1007/s40820-026-02348-9

**Published:** 2026-10-07

**Authors:** Xiaojuan Cao, Yao Chen, Yongkang Tang, Hong Tao, Songzhan Li, Akhtam Amonov, Guojia Fang, Fang Yao

**Affiliations:** 1 School of Electronics and Electrical Engineering, and State Key Laboratory of New Textile Materials and Advanced Processing, Wuhan Textile University, Wuhan, 430200 People’s Republic of China; 2 Key Laboratory of Artificial Micro/Nano Structures of Ministry of Education, School of Physics and Technology, Wuhan University, Wuhan, 430072 People’s Republic of China; 3 Hubei Key Laboratory of Plasma Chemistry and Advanced Materials, Wuhan Institute of Technology, 206 Guanggu 1 st Road, Wuhan, 430205 People’s Republic of China; 4 Department of Optics and Spectroscopy, Engineering Physics Institute, Samarkand State University, University boulevard 15, 140104 Samarkand, Uzbekistan

**Keywords:** Self-assembled monolayer, Interface, Carrier injection, Exciton quenching, Perovskite light-emitting diodes

## Abstract

Molecular interface engineering has become increasingly important for improving the efficiency and stability of perovskite light-emitting diodes (PeLEDs). Although highly emissive perovskite materials have advanced rapidly, their translation into high-performance devices remains limited by interfacial losses, unbalanced charge injection, and instability under electrical operation. In this review, we first discuss charge-carrier behavior in perovskite materials and devices, with emphasis on radiative and non-radiative recombination, carrier accumulation under injection, and the device-level consequences of interfacial constraints. We then examine the development of interfacial molecular strategies in PeLEDs, including self-assembled monolayers and related interfacial molecules, and summarize how such molecular layers influence charge injection, interfacial recombination, local electronic structure, and film formation at both charge-injection interfaces. On this basis, we further consider the implications of these interfacial effects for molecular regulation in light-emitting devices, with particular attention to electronic regulation under bias, chemically persistent passivation, and structural compatibility with emissive-layer formation. By connecting carrier physics, interfacial function, and molecular regulation, this review aims to provide a clearer framework for understanding and developing molecular interfacial strategies for high-performance PeLEDs.

## Highlights

- Establishes a carrier-behavior-driven framework linking high-injection recombination, interfacial carrier crowding, and efficiency roll-off in perovskite light-emitting diodes (PeLEDs).
- Reveals how interfacial molecules, with self-assembled monolayers as a representative class, regulate charge balance, interfacial recombination, local electronic structure, and bottom-interface crystallization.
- Distills practical molecular design principles for high-performance PeLEDs, with emphasis on electronic regulation under bias, persistent passivation, and structural compatibility.

## Graphical abstract

**Figure:**
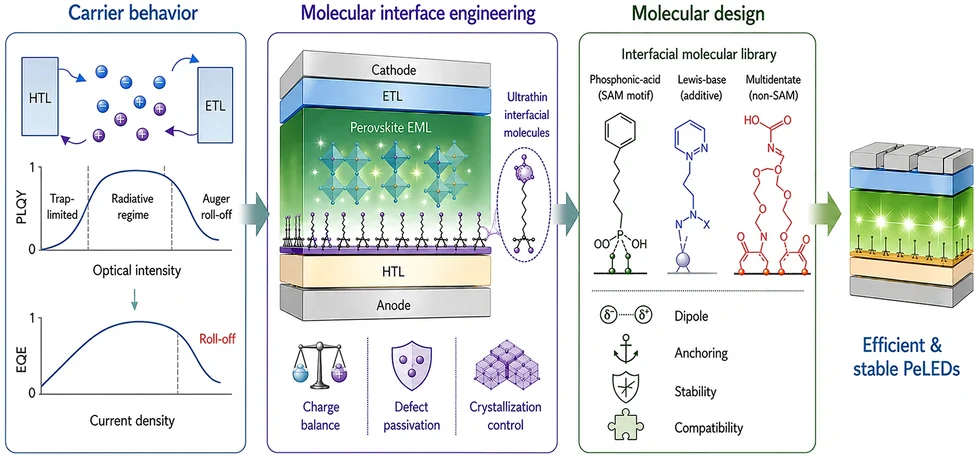

## Introduction

Metal halide perovskites have emerged as a versatile class of semiconductors for light‐emitting diodes, owing to their high photoluminescence quantum yields (PLQYs), defect tolerance, and solution processability [1–8]. Over the past decade, perovskite light‐emitting diodes (PeLEDs) have experienced remarkable progress, with reported external quantum efficiencies (EQEs) surpassing 30% in red devices, 42% in green devices, and 25% in blue devices [9–17]. These features also make PeLEDs attractive for a range of applications, particularly in high-color-purity displays and advanced lighting. In addition, their solution processability, spectral tunability, and compatibility with thin-film device platforms have stimulated growing interest in emerging applications such as optical communication and integrated optoelectronic devices [4, 18, 19]. Even so, further progress in efficiency, operational stability, and spectral purity remains strongly limited by interfacial losses.

This issue is closely related to the carrier environment under which PeLEDs operate. Under electrical injection, carrier densities in PeLEDs can reach 10^17^−10^18^ cm^−3^, far exceeding those typically encountered in perovskite solar cells (PSCs) under 1-sun illumination (below 10^16^ cm^−3^) [20–25]. Under such conditions, radiative recombination competes directly with trap-assisted loss, carrier accumulation, and high-density non-radiative processes [26–29]. As a result, device performance is governed not only by the intrinsic emissive quality of the perovskite layer, but also by how effectively the full device structure controls carrier injection, transport, and recombination under bias. Increasing evidence suggests that interfaces between the perovskite emissive layer and charge-transport layers play a decisive role in efficiency roll-off and long-term instability in PeLEDs [27, 30–34].

A range of strategies has been developed to mitigate these interfacial losses, including bulk additives, surface passivation molecules, polymeric interlayers, and compositional engineering of transport layers [19, 35–40]. While these approaches have delivered incremental improvements, they often suffer from intrinsic limitations, such as molecular migration, uncontrolled thickness, energetic disorder, and parasitic optical absorption. These drawbacks are particularly detrimental for PeLEDs, where exciton quenching and charge imbalance at interfaces can rapidly suppress radiative recombination [41, 42]. Among the molecular strategies developed for this purpose, self-assembled monolayers (SAMs) have attracted increasing attention. Owing to their molecularly defined thickness, ordered packing, and tunable terminal functionality, SAMs offer a distinctive means of regulating interfacial properties with minimal geometric and optical penalty [43–46]. These characteristics are particularly attractive in light-emitting devices, where the interfacial layer ideally remains ultrathin while still exerting a measurable electronic effect. At the same time, SAMs represent only one part of a broader class of interfacial molecules that can influence PeLED operation. More generally, molecular layers at charge-injection interfaces can modify interfacial energetics, alter local electronic structure, affect carrier-selective injection, and reshape the conditions under which radiative recombination occurs in the adjacent perovskite region.

**Fig. 1:**
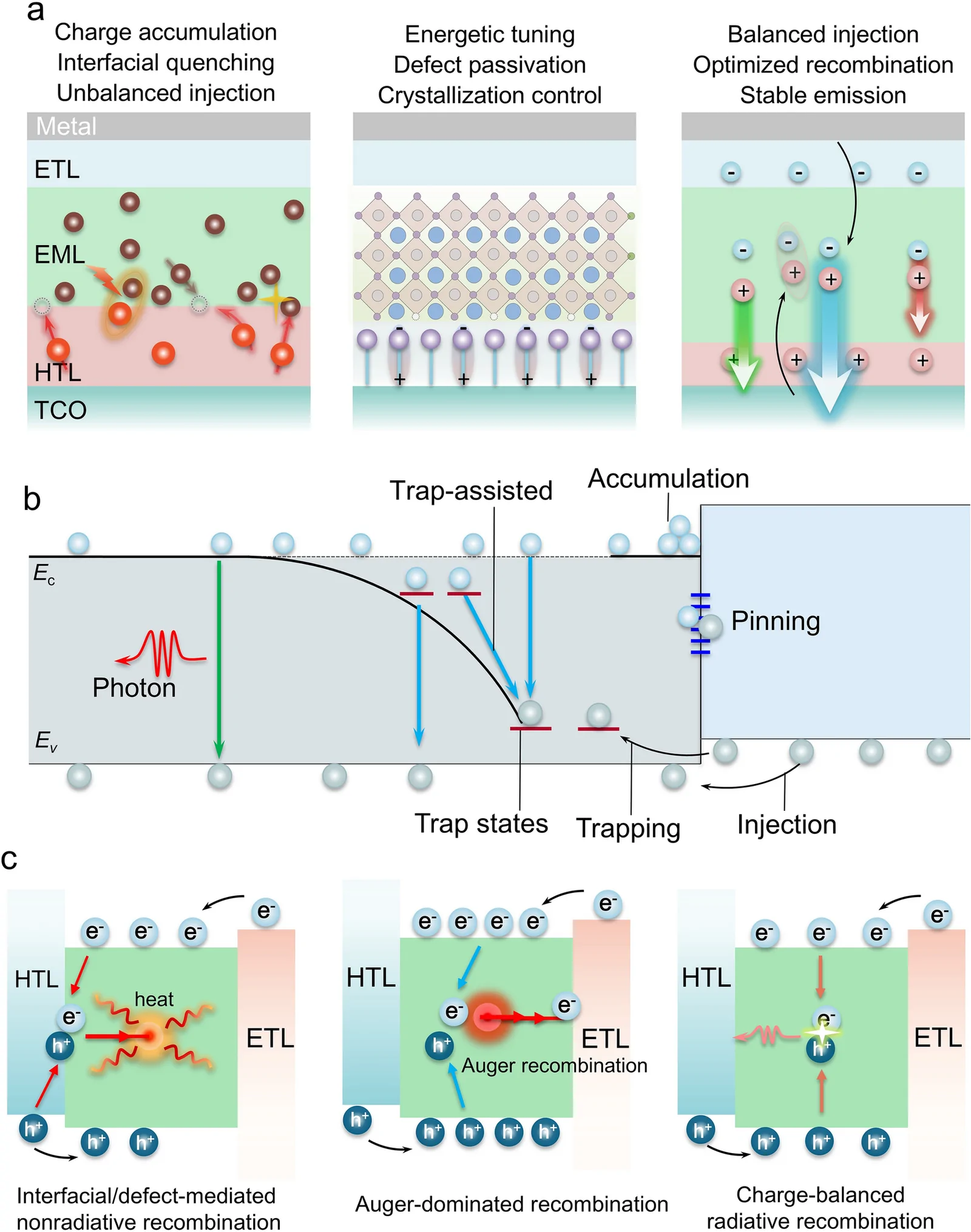
**a** Schematic illustration of interface-limited device operation and molecular interfacial regulation in PeLEDs. Under electrical operation, charge accumulation, interfacial quenching, and unbalanced injection can develop at charge-injection interfaces. Interfacial molecules, including self-assembled monolayers and related ultrathin molecular layers, can regulate interfacial energetics, passivate defects, and influence crystallization near the contact, thereby promoting balanced injection, optimized recombination, and stable emission. **b** Energy-band diagram illustrating interfacial recombination and trapping mechanisms in PeLEDs. Interfacial trap states induce Fermi-level pinning, carrier trapping, and trap-assisted non-radiative recombination, accompanied by carrier accumulation near the injection interface. **c** Schematic illustration of recombination outcomes at buried interfaces in PeLEDs under electrical injection. Poorly regulated interfaces lead to interfacial/defect-mediated non-radiative recombination, excessive carrier buildup under high injection promotes Auger-dominated recombination, whereas balanced electron and hole injection favors efficient radiative recombination

These coupled relationships are summarized schematically in Fig. 1. Figure 1a outlines the overall device picture, in which unbalanced injection, local carrier accumulation, and interface-related non-radiative loss emerge at the charge-injection interfaces under electrical operation, while interfacial molecules can regulate these processes through electronic, chemical, and structural effects. Figure 1b highlights the energetic origin of interfacial loss, including trap-assisted recombination, carrier trapping, and Fermi-level pinning at the contact region. Figure 1c further makes the device mechanism under electrical bias more explicit. In an operating PeLED, electrons are injected from the cathode side and transported through the electron-transport layer, while holes are injected from the anode side and transported through the hole-transport layer. Under bias, the injected carriers establish a recombination zone in the emissive layer, where electron–hole recombination leads to photon emission. On this basis, Fig. 1c illustrates three representative recombination scenarios. Poorly regulated interfaces can lead to interfacial/defect-mediated non-radiative recombination, whereas strong local carrier crowding under unbalanced injection can drive Auger-dominated recombination. By contrast, when interfacial regulation improves charge balance and suppresses interfacial loss, the device is shifted toward charge-balanced radiative recombination. In this sense, the role of interfacial molecules in PeLEDs should not be viewed only through static energy-level alignment, but through their consequences for carrier behavior and recombination under bias.

Despite the rapid growth of this area, current discussions remain somewhat fragmented. Many studies are still organized around individual molecular systems or isolated device improvements, while broader summaries often emphasize general interfacial functions without fully connecting them to the operating requirements of light-emitting devices. In particular, the links between carrier behavior in emissive perovskites, interface-limited device operation, and the molecular regulation of charge-injection interfaces remain insufficiently integrated. This gap becomes more significant as PeLEDs move toward higher brightness, narrower spectral targets, and more demanding stability requirements.

In this review, we summarize recent progress in molecular interface engineering in PeLEDs from this broader perspective, with self-assembled monolayers as an important representative class. We begin with charge-carrier behavior in perovskite materials and devices, focusing on recombination processes, carrier accumulation under injection, and the device-level consequences of interfacial loss, as shown in Fig. 1. We then examine the development of interfacial molecular strategies in PeLEDs and summarize how such molecular layers influence charge injection, interfacial recombination, local electronic characteristics, and film formation at relevant interfaces. On this basis, we further consider the implications of these effects for molecular regulation in light-emitting devices, with attention to practically relevant factors such as electronic regulation under bias, persistent passivation, and structural compatibility with emissive-layer formation. Through this progression, the manuscript aims to provide a clearer framework for understanding how interfacial molecules shape PeLED operation and how such effects may be used to guide future device development.

## Carrier Behavior in Perovskite Materials and Devices

Understanding charge accumulation under high injection in PeLEDs first requires a discussion of recombination in the emissive layer itself. In perovskite light-emitting systems, direct evaluation of electroluminescence quantum yield under operating conditions is often non-trivial, and the radiative mechanism is therefore commonly discussed from the perspective of PLQY. In this sense, PLQY is not merely an optical metric, but a practical measure of how efficiently radiative recombination competes against trap-assisted and many-body non-radiative decay over different excitation-density regimes. A key point here is that perovskite emitters cannot be classified simply as 3D versus 2D, because practical PeLED systems span a broader range of confinement regimes. In bulk-like or weakly confined perovskites, where free carriers are the dominant photoexcited species, radiative recombination is commonly treated as a second-order process competing with first-order trap-assisted recombination and third-order Auger recombination [47–49]. By contrast, in strongly confined low-dimensional systems, including excitonic quasi-2D phases, nanocrystals, and related emitters, radiative decay is more appropriately described as excitonic recombination in competition with trap-assisted exciton loss and exciton–exciton annihilation [21, 50, 51]. This distinction is essential, because it means that the density dependence of luminescence must be interpreted differently for free-carrier-dominated and exciton-dominated systems. On this basis, this section first outlines the major recombination pathways and their carrier-density dependence, and then discusses how carrier density shapes the luminescence behavior of perovskite films, and finally examines how these density-dependent processes manifest in operating devices under high injection.

### Carrier Density, Recombination, and Luminescence in Perovskites

**Fig. 2:**
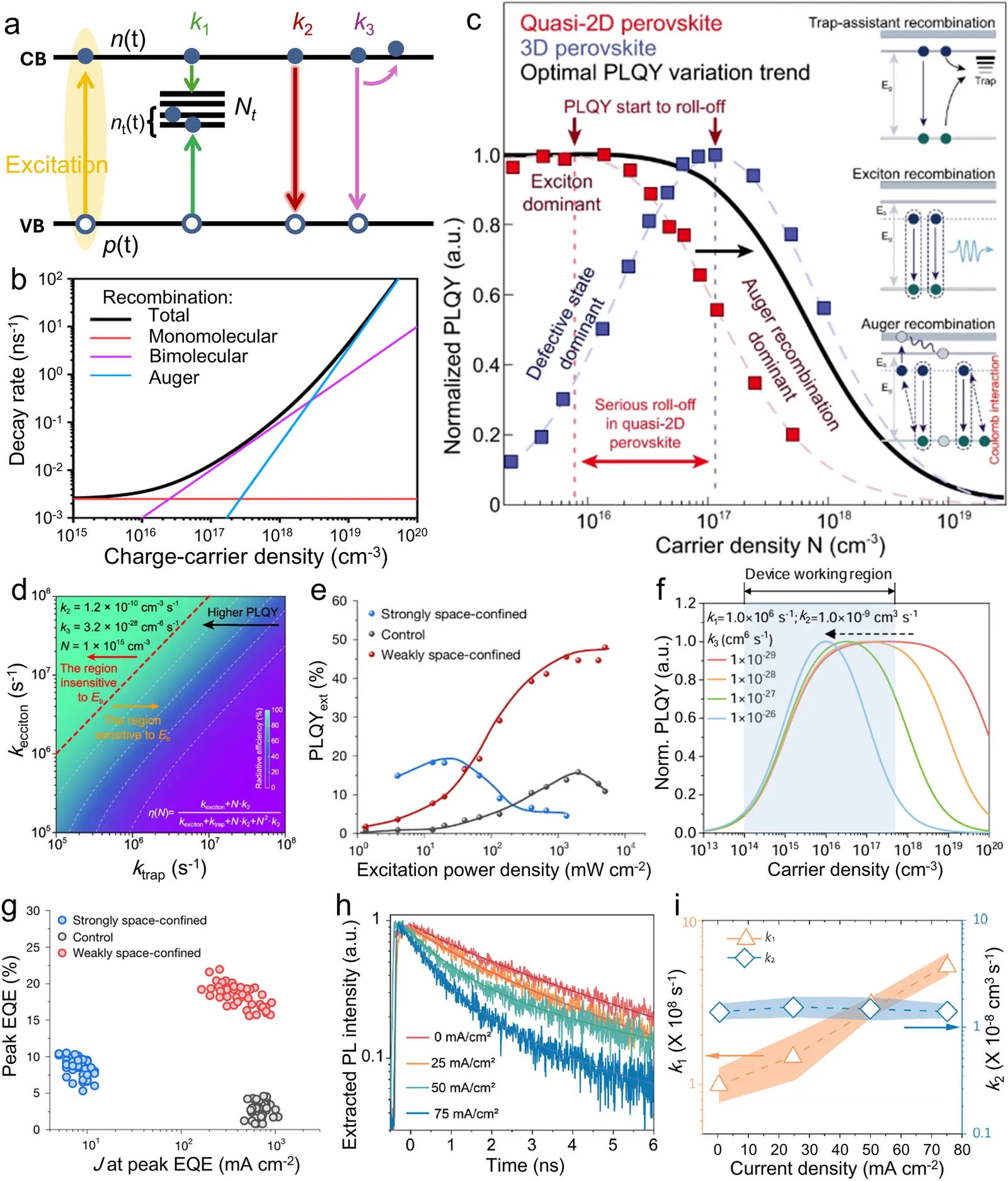
**a** Schematic illustration of carrier relaxation pathways in perovskites following excitation. The total recombination rate is governed by three competing channels: trap-assisted monomolecular recombination (rate constant *k*_1_) via trap states (*N*_t_, trap density *n*_t_(*t*)), bimolecular radiative recombination (*k*_2_), and three-body Auger recombination (*k*_3_). The balance among these processes determines the evolution of carrier populations *n*(*t*) and *p*(*t*).** b** Decay rates of different recombination channels as a function of charge-carrier density [23]. At low carrier densities, monomolecular trap-assisted recombination dominates; at intermediate densities, bimolecular radiative recombination becomes the primary pathway; and at high densities, Auger recombination increases sharply, ultimately limiting radiative efficiency. Copyright 2024, IOP Publishing. **c** Comparison of normalized PLQY trends for quasi-2D and 3D perovskites. **d** Calculated PLQY map illustrating the coupled influence of trap-assisted and many-body non-radiative loss terms on the high-PLQY regime [47]. Copyright 2021, Nature Publishing Group.** e** Experimental PLQY as a function of excitation power density for strongly space-confined, control, and weakly space-confined perovskite emitters. **f** Simulated normalized PLQY as a function of carrier density for different *k*_3_ values. **g** Corresponding relationship between peak EQE and the current density at which peak EQE is reached [54]. Copyright 2025, Nature Publishing Group.** h** Current-dependent extracted photoluminescence decay in an operating PeLED. **i** Current dependence of the extracted recombination coefficients [48]. Copyright 2024, American Physical Society

As illustrated in Fig. 2a, the luminescence of perovskite emitters is governed not by a single decay pathway, but by a competition among several recombination channels that become active over different carrier-density regimes. Following photoexcitation or electrical injection, electrons and holes can recombine radiatively, be lost through trap-assisted non-radiative processes, or, once the carrier population becomes sufficiently high, undergo many-body non-radiative decay [52, 53]. This competition is commonly described by three kinetic terms: first-order recombination (*k*_1_), second-order recombination (*k*_2_), and third-order recombination (*k*_3_). Here, *k*_1_ denotes the first-order monomolecular loss coefficient, which is typically dominated by trap-assisted non-radiative recombination and may also include other defect- or interface-mediated monomolecular losses; *k*_2_ is the bimolecular radiative recombination coefficient for electron–hole pairs; and *k*_3_ is the third-order Auger coefficient, describing non-radiative energy transfer from a recombining electron–hole pair to a third carrier. For free-carrier-dominated systems, this competition is commonly described by *k*_1_, *k*_2,_ *k*_3,_ respectively, so that the carrier dynamics can be written as [23]:

1 $$- \frac{{{\mathrm{d}}n}}{{{\mathrm{d}}t}} = k_{1} n + k_{2} n^{2} + k_{3} n^{3}$$

and the corresponding PLQY as:

2 $${\mathrm{PLQY}} = \frac{{k_{2} n}}{{k_{1} + k_{2} n + k_{3} n^{2} }}$$

where *n* is the free-carrier density. In this expression, the denominator represents the total carrier-decay rate, whereas the numerator corresponds to the radiative part. The relative magnitudes of these terms therefore determine whether the system is trap-limited, radiatively efficient, or already entering the high-density roll-off regime. In exciton-dominated low-dimensional systems, an analogous description can be written as:

3 $$- \frac{{{\mathrm{d}}N}}{{{\mathrm{d}}t}} = (k_{{{\mathrm{trap}}}} + k_{{{\mathrm{ex}}}} )N + k_{{\text{ex - ex}}} N^{2}$$

with

4 $${\mathrm{PLQY}} = \frac{{k_{{{\mathrm{ex}}}} }}{{(k_{{{\mathrm{trap}}}} + k_{{{\mathrm{ex}}}} ) + k_{{\text{ex - ex}}} N}}$$

where *N* is the exciton density, and *k*_trap_, *k*_ex_, and *k*_ex-ex_ denote trap-assisted exciton loss, radiative exciton recombination, and exciton-exciton annihilation, respectively. More specifically, *k*_trap_ describes first-order non-radiative decay of excitons into traps or dark states, *k*_ex_ is the radiative decay rate of excitons, and *k*_ex-ex_ quantifies the bimolecular annihilation of two excitons at elevated excitation density. In this regime, the low-density competition is mainly between *k*_trap_ and* k*_ex_, whereas high-density quenching is introduced by the *k*_ex-ex_*N*^2^ term. Framed in this way, PLQY is not a fixed material constant, but the outcome of competition among radiative and non-radiative channels over different density regimes. The central question is therefore not simply whether a perovskite film is emissive, but which recombination channel dominates at a given carrier density and how wide the density window remains in which radiative recombination can prevail.

Figure 2b further highlights why this framework is central to PeLEDs. The three channels do not scale equally with carrier density: At low density, first-order loss dominates because trap-assisted recombination competes effectively with light emission; as the carrier density increases, radiative recombination becomes more competitive and the emissive layer enters a regime favorable for high luminescence; once the density rises further, however, the third-order Auger term increases rapidly and eventually overtakes the radiative channel, leading to luminescence quenching. The significance of the *k*_1_- *k*_2_- *k*_3_ model therefore lies in the fact that each term points to a different material or interfacial limitation. A large *k*_1_ usually indicates insufficient defect suppression, and a favorable *k*_2_ reflects efficient electron–hole overlap and radiative recombination, whereas a rapidly increasing *k*_3_ signals that the material will become vulnerable to efficiency roll-off under high injection.

This carrier-density dependence becomes particularly consequential when different perovskite dimensionalities are compared. As summarized in Fig. 2c, the carrier-density window over which luminescence remains optimal is not universal, but shifts with the degree of confinement. In quasi-2D perovskites, PLQY rises rapidly at low carrier density because exciton confinement and energy funneling can make radiative recombination highly efficient once shallow defects are saturated. However, the same features also compress the usable density window: The local carrier population at the emissive phase builds up more quickly, so the onset of PLQY roll-off occurs at comparatively lower carrier density and the high-density regime becomes Auger-dominated earlier. For example, Jiang and co-workers provided a representative example of how reducing *k*_3_ can widen the useful operating range of quasi-2D perovskites, as shown in Fig. 2d [47]. By lowering the exciton binding energy, they suppressed Auger recombination by more than one order of magnitude and increased the PLQY roll-off threshold by about fivefold. The same trend was reflected in device performance, with *J*_90_ increasing from 15.9 to 82.6 mA cm^−2^, together with a peak EQE of 20.36% and a maximum luminance of 82,480 cd m^−2^. Sun and co-workers showed that *n*=3 films can reach a peak PLQY of 91% at an excitation density of about 6.0×10^15^ cm^−3^, with little decline below 10^16^ cm^−3^, but that all compositions eventually exhibit PLQY roll-off as excitation density increases [55]. Importantly, the roll-off threshold increased with increasing *n*, indicating that less strongly confined systems can better tolerate high carrier densities even if their peak PLQY is not the highest. Yu and co-workers further showed that the radiatively favorable density window in quasi-2D perovskites is highly sensitive to phase evolution itself rather than to nominal confinement alone [56]. In their pure-red system, phase-transition engineering suppressed the *n* = 1 phase while rendering the *n* = 2 and *n* = 3 phases more pronounced, indicating a redistribution from low-n phases toward a more favorable emissive landscape. The optimized devices reached a peak EQE of 25.3%, further showing that control over phase dimensionality and phase purity is directly relevant to sustaining efficient recombination under device-relevant carrier densities. Zheng and co-workers provided a complementary device-side example from the perspective of band alignment engineering in quasi-2D LEDs [57]. By tailoring the energetic relationship among confined domains, they showed that carrier dynamics can be reshaped not only through intrinsic confinement itself, but also through the local band structure established across the quasi-2D landscape. The resulting devices reached 1000 cd m^−2^ at a low driving voltage of 2.8 V and exhibited a record *T*_50_ of 318.8 min at 1000 cd m^−2^, consistent with the view that once phase distribution and band alignment are simultaneously improved, high-density recombination loss and operational instability can both be alleviated.

By contrast, 3D perovskites generally tolerate a broader carrier-density range before severe luminescence quenching sets in, even though their PLQY increase at low density may be less abrupt. Peng and co-workers further showed that weakly space-confined all-inorganic perovskites can simultaneously retain high carrier mobility and reduced Auger loss [54]. This trend is reflected in Fig. 2e, where the weakly space-confined emitter maintains a broader excitation-density range favorable for efficient emission and reaches a markedly higher PLQY than the strongly space-confined and control samples at high excitation power density. Li and co-workers showed that electron-withdrawing trifluoroacetate anions in 3D perovskite emitters can retard Auger recombination [58]. As a result, the devices maintained an external quantum efficiency above 20% even at current densities as high as 2270 mA cm^−2^. This result indicates that delaying high-density Auger loss can substantially widen the radiatively favorable carrier-density window. Chen and co-workers provided a complementary example from the low-density side of the recombination landscape [59]. By using an interfacial amination reaction, they prepared FAPbBr_3_ films with an ultralow trap density of 1.2×10^10^ cm^−3^, thereby improving the low-density luminescence regime by suppressing first-order loss.

In this context, Fig. 2f emphasizes the role of *k*_3_ within the carrier-density range relevant to device operation. The key issue is not simply that Auger recombination will eventually dominate at sufficiently high density, but whether it begins to impose a significant penalty already within the working regime of a PeLED. A smaller *k*_3_ shifts the onset of PLQY decay to higher carrier density and broadens the density interval over which efficient emission can be sustained during device operation, whereas a larger *k*_3_ causes the emissive layer to enter the roll-off regime prematurely. In this sense, *k*_3_ determines how much practical operating margin exists before high-injection loss becomes severe. As a result, density-dependent film luminescence is not just a photophysical characterization exercise; it is a practical predictor of whether a given emitter will remain efficient when translated into a working device.

### Charge Injection and Accumulation

Under electrical bias, injection barriers, transport asymmetry, and interfacial defect states can drive electrons and holes to accumulate within a spatially confined recombination zone, so that locally high carrier densities may develop even when the average carrier population in the emissive layer is not yet extreme. In this regime, device performance is governed not only by the intrinsic radiative efficiency of the perovskite film, but also by how effectively the operating structure distributes carriers and mitigates interfacial loss channels.

In operating PeLEDs, such local carrier crowding is frequently manifested as pronounced EQE roll-off at elevated current densities. This point is also reflected in Fig. 2g, where the current density at peak EQE differs markedly among emitters with different confinement characteristics. In particular, the weakly space-confined system reported by Peng et al. reaches its peak efficiency at substantially higher current density than the strongly space-confined and control systems, while also maintaining a much higher peak EQE [54]. Zou et al. reported that efficiency loss at high current density in layered perovskite LEDs was closely associated with luminescence quenching, likely arising from non-radiative Auger recombination [60]. By tuning the quantum-well width, they achieved a peak EQE of 12.7% and retained ~10% EQE at 500 mA cm^−2^. Jiang *et al.* likewise addressed high-density recombination from the perspective of exciton binding energy, showing that reducing the impact of Auger recombination in quasi-2D perovskites enabled a peak EQE of 20.36% together with a luminance of 82,480 cd m⁻^2^, indicative of substantially suppressed roll-off at high drive levels [47]. Fakharuddin *et al.* approached the same problem from the device side and showed that balancing electron and hole injection in mixed 2D/3D PeLEDs led to a nearly flat EQE over a wide current range, together with a marked improvement in operational lifetime [61].

The dynamical origin of this roll-off can also be followed directly under operation. As shown in Fig. 2h, the extracted luminescence decay becomes progressively faster as the driving current density increases, indicating that the recombination dynamics shift systematically once the device enters the high-injection regime. Consistent with this trend, Fig. 2i shows that the first-order loss coefficient increases with current density, whereas the radiative coefficient remains comparatively less sensitive [48]. This behavior suggests that current-dependent efficiency loss is associated not simply with a higher carrier population, but with the growing importance of non-radiative channels under electrical loading. These measurements therefore provide a direct link between EQE roll-off and the evolution of recombination kinetics in operating devices. The current-dependent extracted photoluminescence decay and recombination coefficients shown in Fig. 2h, i were reported by Zhang and co-workers using in situ ultrafast spectroscopy on working PeLEDs.

**Fig. 3:**
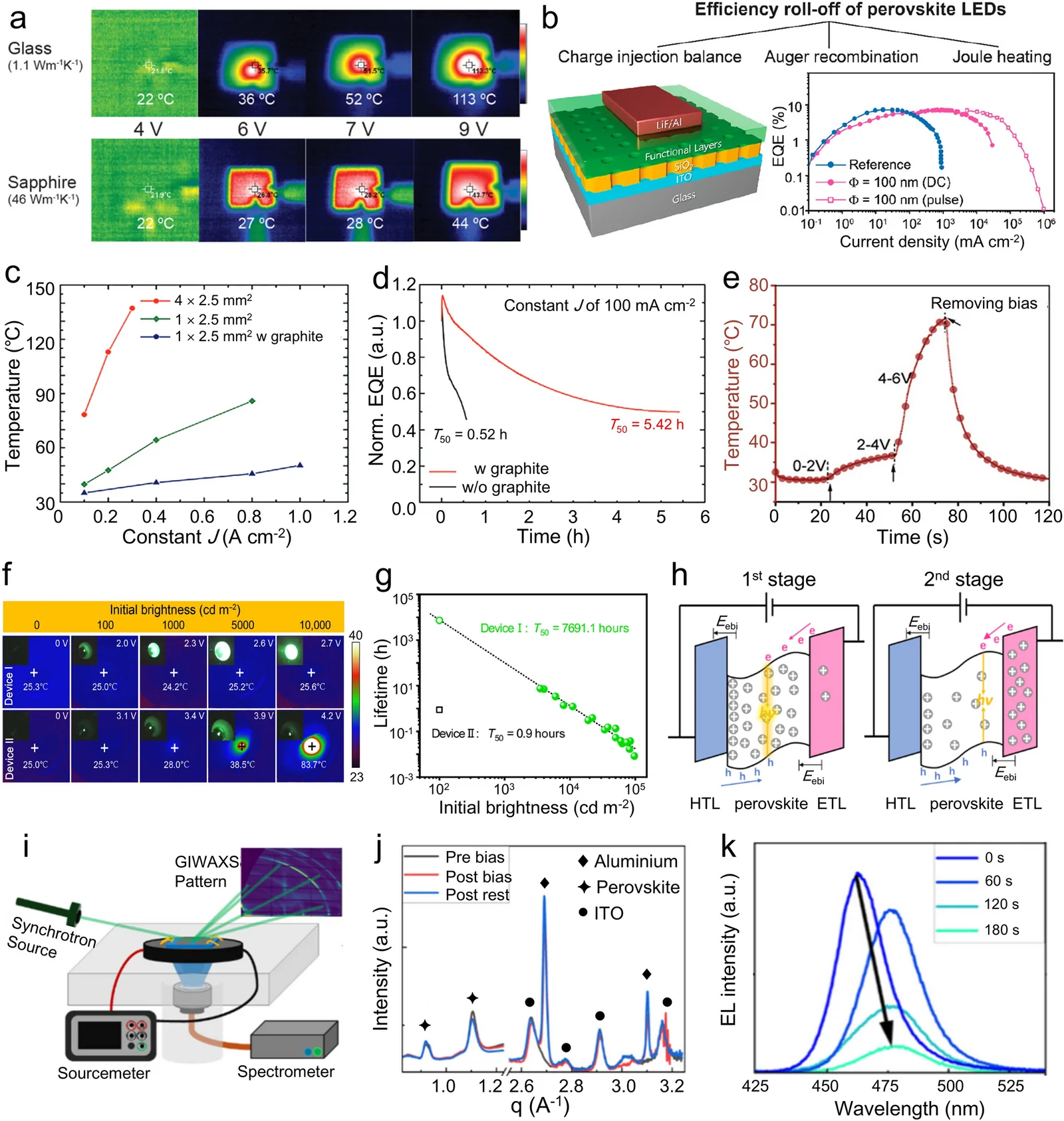
**a** Infrared thermal images of PeLEDs fabricated on glass and sapphire substrates under increasing bias.** b** Coupled origins of efficiency roll-off in PeLEDs and the corresponding EQE-current density characteristics under different thermal-management conditions [62]. Copyright 2020, American Chemical Society.** c** Device temperature as a function of constant current density for different device sizes and thermal-management configurations.** d** Normalized EQE decay at a constant current density of 100 mA cm^−2^ for devices with and without graphite heat spreading [63].** e** Transient temperature evolution under stepwise increasing bias and after bias removal [64]. Copyright 2020, Wiley-VCH. **f** Infrared thermal images of Device I and Device II recorded at different initial brightness values. **g** Lifetime-brightness relationship of Device I and Device II. Copyright 2024, AAAS.** h** Schematic illustration of the proposed two-stage degradation process in quasi-2D PeLEDs [66]. Copyright 2020, American Chemical Society.** i** Operando measurement configuration combining GIWAXS with simultaneous electrical and spectroscopic monitoring for tracking structural evolution in working deep-blue PeLEDs under bias. **j** Operando GIWAXS profiles collected before bias, after bias, and after rest.** k** Time-dependent electroluminescence spectra during device operation [67]. Copyright 2025, American Chemical Society

As the drive level increases further, the same electrical conditions that compress the recombination zone also aggravate Joule heating, particularly when injection barriers, resistive transport layers, or limited thermal dissipation raise the local device temperature. Zou and co-workers directly visualized the temperature distribution of operating PeLEDs on different substrates [62]. Fig. 3a shows that devices on glass underwent a much stronger temperature rise than corresponding devices on sapphire under the same bias, highlighting the role of thermal dissipation in limiting high-current operation. By combining improved charge-injection balance, reduced Auger loss, sapphire substrates, and current focusing, the authors extended the usable current-density range and suppressed thermally amplified roll-off (Fig. 3b), enabling green PeLEDs to operate at current densities approaching ~ 1 kA cm^−2^ and achieved a record luminance of 7.6 × 10^6^ cd m^−2^. Zhao et al. further showed that thermal accumulation strongly affects EQE roll-off under high-current operation [63]. A 4 × 2.5 mm^2^ glass device reached ~ 140 °C at ~ 300 mA cm^−2^, whereas a 1 × 2.5 mm^2^ device with graphite remained near 50 °C even at ~ 1000 mA cm^−2^, as shown in Fig. 3c. Correspondingly, the 1 × 2.5 mm^2^ sapphire device with graphite maintained EQE above 10% up to ~ 2000 mA cm^−2^, together with a peak EQE of 17% and an approximately one-order-of-magnitude improvement in lifetime at 100 mA cm^−2^ (Fig. 3d). These results show that suppressing thermal accumulation can substantially widen the high-current operating window of PeLEDs. In a related thermal study, Zou et al. showed that Joule heating can directly drive interfacial degradation during PeLED operation [64]. Under stepwise increasing bias, the device temperature increased from ~ 30 to ~ 70 °C and dropped rapidly after the bias was removed, indicating clear thermal accumulation under electrical driving (Fig. 3e). The authors attributed the resulting performance decay to Joule-heating-induced morphological evolution of TPBi, which promoted elemental interdiffusion at the contact and caused photoluminescence quenching. More recently, Zheng et al. showed that PeLED stability can be markedly improved by suppressing Joule heating through ultralow-voltage operation [65]. Their optimized green PeLED with the structure ITO/poly-TPD/Me-4PACz:PVP/core-shell perovskite NCs/PO-T2T/LiF/Al (Device I) achieved an ultralow turn-on voltage of 1.7 V, a peak EQE of 27.8%, and a maximum brightness of 90,295 cd m^−2^. As reflected in Fig. 3f, Device I exhibited only a negligible temperature rise from 25.3 to 25.6 °C as the brightness increased from 0 to 10,000 cd m^−2^, whereas the control device (Device II) showed a much larger increase from 25.0 to 83.7 °C over the same brightness range. Correspondingly, the lifetime-brightness relationship in Fig. 3g showed a dramatic stability advantage, with Device I reaching a *T*_50_ of 7691.1 h, compared with only 0.9 h for Device II. These results indicate that reducing interfacial recombination and lowering the operating voltage can effectively suppress heat accumulation, thereby substantially improving both high-brightness operation and long-term stability in PeLEDs.

Under sustained operation, carrier crowding and temperature rise can in turn facilitate ion redistribution and interfacial chemical evolution, making high-injection loss and degradation difficult to separate in practice. Cheng et al. linked high-injection degradation in quasi-2D PeLEDs to ligand–cation migration through domain boundaries [66]. They proposed a two-stage process: an initial stage in which cation redistribution weakens defect passivation and causes reversible EQE decay, followed by a second stage in which continued migration brings mobile species into contact with adjacent transport layers, resulting in stronger interfacial non-radiative loss and irreversible degradation (Fig. 3h). Operando measurements by Mirabelli et al. further showed that failure in deep-blue PeLEDs is closely associated with chemical evolution at the electron-injecting interface rather than with immediate bulk structural collapse [67]. As shown in Fig. 3i, the authors combined operando GIWAXS with electroluminescence measurements to track structural and spectral evolution under bias. The diffraction features of the perovskite layer changed only modestly after bias and partially recovered after rest (Fig. 3j), whereas the EL spectrum showed a pronounced red shift and intensity loss within 180 s of operation (Fig. 3k). These results indicate that device degradation was dominated by interfacial chemical changes under bias, even though the bulk perovskite framework remained comparatively intact.

These material-level photophysical characteristics provide the necessary basis for understanding PeLED operation, but they do not by themselves determine device performance. Parameters derived from PLQY, TRPL, and carrier-density-dependent luminescence measurements indicate how efficiently radiative recombination competes with trap-assisted and high-density non-radiative loss in the emissive material. Once the material is incorporated into a working device, however, the carrier population is no longer governed only by intrinsic recombination kinetics, but also by how charges are injected, accumulated, and redistributed at the two charge-injection interfaces under bias. In this sense, the translation from film-level emissive properties to device-level efficiency and stability is mediated by the interface. This is precisely why interfacial molecular regulation becomes essential in PeLEDs: It determines whether the favorable carrier-recombination characteristics identified at the material level can be maintained under actual electrical operation.

## Development and Emerging Roles of Interface Molecules in PeLEDs

In PeLEDs, high emissive quality of the perovskite layer alone is not sufficient to ensure efficient and stable device operation. Once electrical injection is involved, the charge-injection interfaces become key regions where carrier distribution, recombination behavior, and operational degradation are shaped. This has made interfacial molecular regulation an increasingly important part of PeLED research. At these interfaces, molecular layers can influence charge injection, reduce interfacial non-radiative loss, and modify the local structural and electronic environment adjacent to the perovskite emitter [68–70]. Accordingly, the role of interface molecules in PeLEDs has gradually expanded from simple interfacial modification to a more integral part of device design.

SAMs emerged in this setting as a particularly useful class of interfacial molecules because they can form molecularly thin, ordered, and strongly anchored layers on oxide or semiconductor surfaces. Their early use in PeLEDs was largely driven by several practical advantages, including well-defined thickness, tunable surface energetics, negligible parasitic absorption, and chemically addressable terminal groups [43, 44, 71–74]. These features made SAMs attractive alternatives to less well-defined interlayers, especially in light-emitting devices where interfacial regulation must be achieved without introducing an additional transport barrier or excessive optical loss. At the same time, these advantages do not make SAMs universally ideal interfacial layers. In practical PeLED architectures, their effectiveness may still be limited by incomplete surface coverage, strong dependence on substrate chemistry, poor wettability for subsequent perovskite deposition in some systems, molecular disorder, and limited compatibility with non-oxide or chemically less reactive interfaces. In addition, the stability of SAM-modified contacts under prolonged electrical bias or thermal stress remains an important concern, because interfacial dipoles, anchoring integrity, and local packing order may evolve during operation.

With the accumulation of studies, the role of SAMs in PeLEDs has been understood in a broader way. In addition to modifying wettability or facilitating charge injection, they have been used to influence crystallization near the contact, adjust local defect chemistry, regulate interfacial charge accumulation, and improve device stability under operation. More generally, this development reflects a shift in how interface molecules are viewed in PeLEDs: not simply as auxiliary layers inserted between functional components, but as molecular elements that can shape the structural and electronic environment in regions that are critical to device performance. To provide a more concise overview of this development, Table 1 summarizes representative molecular interfacial layers used in PeLEDs, together with their functional groups, interfacial roles, device structures, and key performance metrics. At the same time, the comparison in Table 1 also shows that the effect of a given SAM cannot be regarded as molecule-intrinsic or transferred directly from one PeLED architecture to another. Even for the same molecular family, the reported device consequences vary substantially with emitter composition, confinement regime, buried-contact configuration, deposition route, and the specific performance metric being evaluated. This point is evident in several frequently used phosphonic-acid carbazole systems. For example, 2PACz has been reported in green, blue, and near-infrared devices, as well as in NiO_x_/PVK-based hole contacts, with its role described variously as molecular packing regulation, defect passivation, interfacial adhesion enhancement, or charge-injection improvement. Br-2PACz likewise shows different consequences in 3D FAPbBr_3_ and quasi-2D bromide systems, where the same molecular motif operates in contact with different emissive phases, transport layers, and carrier-confinement conditions. A similar contrast appears for Me-4PACz: In weakly space-confined all-inorganic green devices, it is associated with efficient high-brightness operation and long projected lifetime, whereas in blue structures its reported gains are much more limited. These differences should not be read simply as contradictory outcomes. Rather, they indicate that the function of an interfacial molecule is expressed through a specific material–interface–device context. Depending on the system, the dominant consequence of the same SAM may lie in work-function tuning, buried-contact reconstruction, wetting and nucleation control, defect suppression, or recombination-zone redistribution.

**Table 1:** Summary of representative molecular interfacial layers and device performance in PeLEDs

	Interfacial molecule	HOMO (eV)	LUMO (eV)	Dipole	Functional group	Device structure	EL_peak_ (nm)	EQE_max_ (%)	*L*_max_ (cd m^−2^)	*V*_on_ (V)	*T*_50_	References
Phosphonic-acid SAMs	2PACz	− 5.81	− 2.01	–	Carbazole, P=O	ITO/Poly-TPD/PVK/2PACz/perovskite/TPBi/LiF/Al	515	33.79	71,000	2.7	75 h@100 cd m^−2^	[75]
							480	15.52	1176	3.0	–	
							804	22.63	50.2 (W sr^−1^ m^−2^)	1.6	–	
	2PACz	− 5.60	–	–	Carbazole, P=O	ITO/2PACz-PVK/perovskite/TPBi/LiF/Al	514	30.4	134,100	2.8	148.4 min @ 100 cd m^−2^	[76]
						ITO/2PACz-PVK/perovskite/TPBi/LiF/Al	485	25.3	4569	3.6	16 min @ 100 cd m^−2^	
	2PACz	− 1.7 vs. NiO_x_	+ 1.7 vs. NiO_x_	–	Carbazole	ITO/NiO_x_/2PACz/PVK/perovskite/TPBi/LiF/Al	493	14.5	10,392	2.1	~1 4 min @ 1 mA cm^−2^	[77]
	2PACz+ MAC	− 5.85	− 3.31	–	Carbazole, P=O	ITO/(PEDOT:PSS/PMMA/perovskite+MAC+2PACz/TPBi/Liq/Al	492	17.1	10,142	3.0	0.28 h @ 4.5 V	[78]
	Me-4PACz	− 5.8	–	–	Carbazole, P=O	ITO/Me-4PACz/PVK/perovskite/B2/LiF/Al	~ 525	22.0	1,167,000	<2	185,600 h @100 cd m^−2^	[54]
	Me-4PACz	− 5.7	− 2.5	–	Carbazole, P=O	ITO/Me-4PACz/PVK/perovskite/TPBi/Liq/Al	458	4.3	59.82	3.7	9.55 min @ 1 mA cm^−2^	[79]
	MeO-2PACz	− 5.3	–	–	–OCH_3,_ carbazole	ITO/PEDOT:PSS/MeO-2PACz/perovskite/TPBi/LiF/Al	513	21.5	17,310	3.1	14.5 h @ 1000 cd m^−2^	[80]
SAM derivatives	I-2PACz	− 5.81	− 2.06	4.56D	Carbazole	ITO/(Cu:NiO+I-2PACz)/TFB/QD/ZnMgO/Al	525	27.0	92,735	2.4	11852 h @ 100 cd m^−2^	[81]
	Br-2PACz	–	–	–	Carbazole	ITO/Br-2PACz/FAPbBr_3_/bilayer ZnO/Ag	540	20.7	312,000	1.9	350 h @ 1000 cd m^−2^	[59]
	Br-2PACz	− 6.0	–	–	Carbazole	ITO/Br-2PACz/PEA_2_Cs_4_Pb_5_Br_16_: C_8_-BTBT/TPBi/Ca/Al	515	18.6	45,276	2.6	18.5 min @ 100 cd m^−2^	[82]
	Me-4PACz:PVP	− 5.65	− 2.31	–	Carbazole, P=O	ITO/poly- TPD/Me- 4PACz:PVP/core- shell perovskite NCs/PO-T2T/LiF/Al	515	27.8	90,295	1.5	7691.1 h@ 100 cd m^−2^	[65]
	Me-4PACz:PVP	− 2.31	− 5.65	–	Carbazole, P=O	ITO)/poly-TPD/Me-4PACz:PVP/perovskite/PO-T2T/LiF/Al	496	21.5	7449	1.9	318.8 min @ 1000 cd m^−2^	[83]
	PFNBr+2PACz	− 5.82	–	–	Br ion and R_4_N^+^ groups	ITO/2PACz/PFNBr/Perovskite/TPBi/LiF/Al	513	12.1	22,121	2.8	23 min @ 150 cd m^−2^	[84]
	S-2PACz	− 5.82	− 1.53	7.1D	S=O, P=O	ITO/S-2PACz/Perovskite/Ga^+^/TPBi/LiF/Al	484	8.6	1944	3.1	0.18 h @ 100 cd m^−2^	[85]
	C-2PACz	− 5.71	− 1.87	6.3D	C=O	ITO/C-2PACz /Perovskite/Ga^+^/TPBi/LiF/Al	484	10.4	1843	~3.0	0.34 h @ 100 cd m^−2^	
Multifunctional molecules	2BrCzPA	− 5.54	–	–		ITO/2BrCzPA/TFB/QDs/ZnMgO/Al	540	15.3	34,250	2.1	360 h @ 1000 cd m^−2^	[86]
	XS10	− 5.37	− 2.53	3.94D	–CN, conjugated alkene unit	ITO/SAM/PTAA/perovskite/TPBi/LiF/Al	~ 520	16.6	33,816	2.6	–	[87]
	MBTPA	− 5.41	–	–	C=O, P=O, –CF_3_	ITO/TFB/PVK/MBTPA/perovskite/TPBi/LiF/Al	485	23.1	4910	3.6	19.2 min @ 100 cd m^−2^	[88]
						ITO/TFB/PVK/MBTPA/perovskite/TPBi/LiF/Al	513	27.1	45,500	3.4	612 min @ 100 cd m^−2^	
	36ClCzEPA	− 6.19	–	–	P=O, Cl	ITO/36ClCzEPA/2D-perovskite/TPBi/LiF/Al	473	4.8	1253	3.0	2.45 min @ 100 cd m^−2^	[89]
	3-GPA (3-guanidinopropionic acid)	–	− 5.28	–	Carboxyl, guanidine	ITO/NiO_*x*_/3-GPA/perovskite/B3PYMPM/LiF/Al	530	25.25	14,612	3.2	93.32 min@0.33 mA cm^−2^	[90]

Direct comparison of absolute EQE or lifetime values across reports therefore requires caution, because device architecture, substrate chemistry, adjacent transport layers, perovskite dimensionality, deposition route, and even the testing protocol can all influence the apparent effectiveness of a molecular layer. The more meaningful question is not whether one SAM universally “outperforms” another, but which interfacial bottleneck it primarily addresses in a given PeLED stack, and under what structural and operational conditions that role becomes decisive. Viewed in this way, Table 1 serves less as a simple ranking of molecules than as a comparative map of molecular functions across different device contexts. This also provides the basis for the discussion below: Before specific molecular effects can be understood, it is first necessary to clarify why PeLED performance becomes so sensitive to the condition of the charge-injection interfaces.

### From Interface Sensitivity to Interface Molecular Adoption in PeLEDs

A characteristic feature of PeLEDs is that radiative recombination is not distributed uniformly across the emissive layer, but is strongly influenced by the condition of the charge-injection interfaces. Under practical operating currents, electrons and holes can accumulate near these contacts, so relatively small interfacial defects or energetic mismatches may have a disproportionate effect on current efficiency, roll-off, and spectral stability. This helps explain why improvements in bulk emissive quality alone have not been sufficient to remove device-level losses.

During the early development of PeLEDs, much attention was directed toward the perovskite emitter itself, including crystallinity, defect density, phase purity, and intrinsic luminescence efficiency. As progress in these areas continued, the factors limiting device performance became increasingly interfacial. The interfaces between the perovskite layer and the adjacent charge-transport layers came to be understood not simply as internal boundaries, but as regions that directly affect charge balance, interfacial recombination, and efficiency loss under electrical operation. This shift was supported by studies showing that suppression of interface-related non-radiative loss or improvement of injection balance can reduce efficiency roll-off and improve operational stability. For example, Xu and co-workers showed that passivation at both top and bottom interfaces was necessary to effectively suppress non-radiative loss [91], whereas Ngai and co-workers linked interfacial injection imbalance to carrier crowding and Auger-related roll-off [92]. Together, these results indicated that the performance limits of PeLEDs are closely associated with interface quality, rather than with bulk emission alone.

**Fig. 4:**
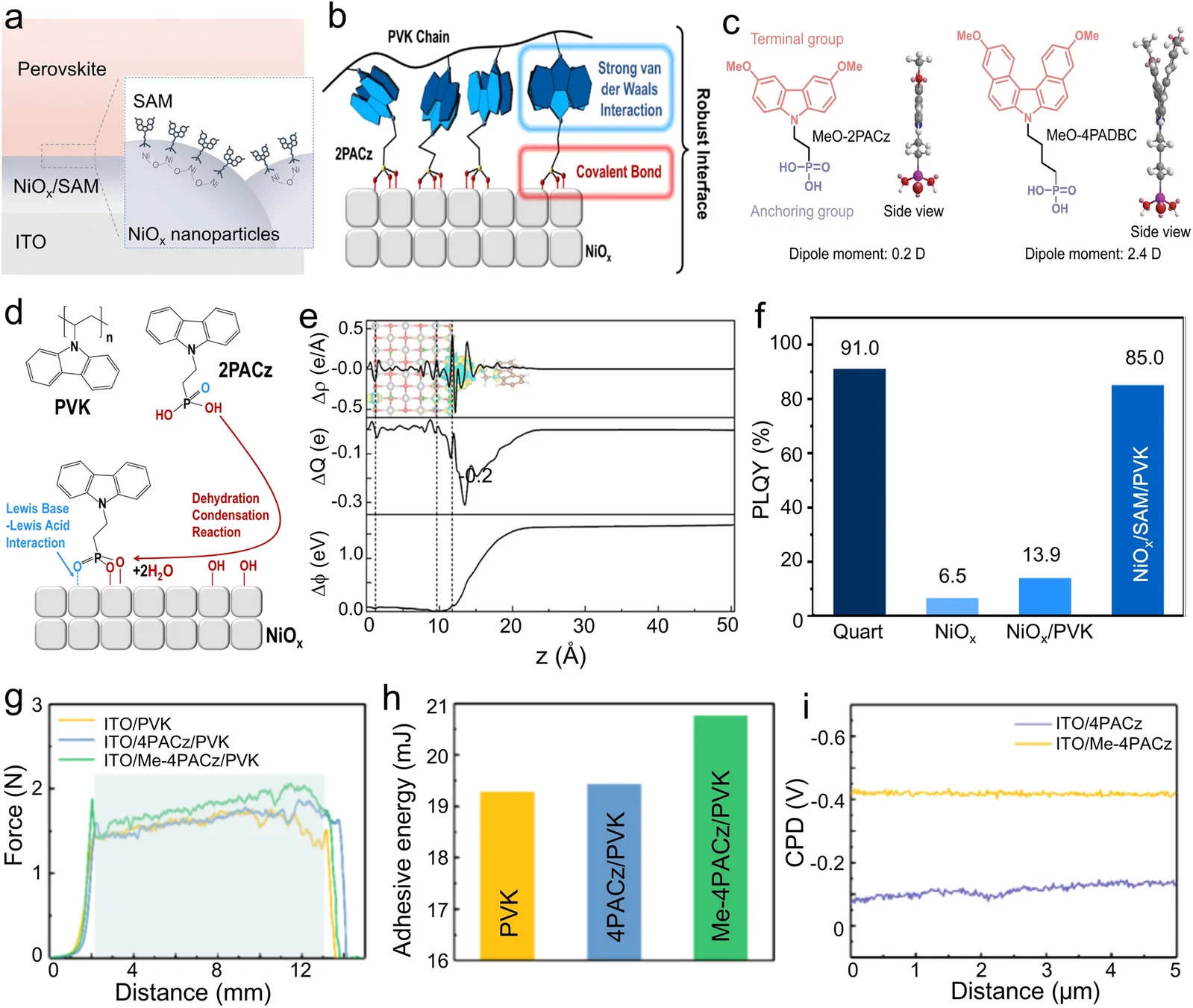
**a** Schematic illustration of a NiO_x_/SAM-modified hole-contact interface in a PeLED [43]. Copyright 2023, AAAS. **b** Schematic representation of 2PACz assembled on a NiO_x_ surface, where the phosphonic acid group anchors to NiO_x_, while the upper aromatic moieties provide interfacial contact with the PVK layer through van der Waals/π-π interactions [77]. Copyright 2023, Nature Publishing Group. **c** Molecular structures, side views, and dipole moments of MeO-2PACz and MeO-4PADCB [43]. Copyright 2023, AAAS.** d** Schematic illustration of the interaction between PVK and the 2PACz-modified NiO_x_ surface. The phosphonic-acid group anchors to NiO_x_ through dehydration condensation, while Lewis acid–base interactions contribute to interfacial coupling. **e** Calculated charge density difference (Δρ), amount of charge transfer (ΔQ) and electrostatic potential step (ΔΦ) at the NiO/SAM interface. **f** PLQY of perovskite films deposited on different substrates [77]. Copyright 2023, Nature Publishing Group.** g** Pull-off force curves for PVK films deposited on bare ITO and SAM-modified ITO.** h** Corresponding adhesive energy extracted from force–distance measurements. **i** Contact potential difference (CPD) measured by Kelvin probe for ITO/4PACz and ITO/Me-4PACz surfaces [93]. Copyright 2024, American Chemical Society

SAMs began to attract attention as a practical strategy for regulating charge-injection interfaces in PeLEDs. Although their earliest and most established use was at oxide-based contacts, related molecular regulation has also been explored at other interfaces, including those involving organic charge-transport layers (Fig. 4a, b) [43, 77]. Compared with conventional interlayers, SAMs offer molecularly defined thickness, ordered packing, and, in suitable systems, strong interfacial anchoring, while avoiding the additional transport distance, optical loss, and structural uncertainty often introduced by thicker layers. Among the SAM systems studied so far, carbazole phosphonic-acid derivatives have been used most extensively, largely because they can form robust monolayers on oxide-containing surfaces and allow interfacial energetics to be adjusted through molecular structure. As a result, molecules such as 4PACz, MeO-4PACz, MeO-4PADCB, and related derivatives became widely used in the construction of hole-injecting contacts (Fig. 4c) [43]. Subsequent studies showed that such SAM-modified interfaces can influence not only peak EQE, but also turn-on behavior, high-brightness efficiency retention, and operational stability.

Their significance in PeLEDs, however, is not limited to energetic regulation alone. Once introduced at a buried contact, these molecular layers also become part of the local environment in which the overlying transport and emissive layers are deposited. The same interfacial modification that alters work function, charge selectivity, or interfacial recombination can therefore also influence wetting, adhesion, local packing, and the conditions under which the perovskite layer first nucleates and develops. From this point onward, the discussion needs to move from the electronic consequences of interface modification to its structural consequences during buried-interface formation.

### Bottom-Interface Crystallization and Film Formation

Beyond their influence on interfacial energetics and charge injection, interface molecules also shape how the buried contact is physically established and how the overlying layers subsequently develop. The interface is not only where charge injection will later occur, but also where the overlying transport and emissive layers first develop. As a result, interface molecules can affect how this contact is formed, including interfacial adhesion, wetting, local packing, and structural order at the bottom interface. In PeLEDs, this effect is especially relevant because the emissive layer is often only tens of nanometers thick, so non-uniformity introduced during bottom-interface formation can readily carry through into carrier confinement, recombination behavior, and overall device performance.

A first effect of interface molecules appears before the perovskite emitter is deposited, namely in the formation of the buried hole-contact itself. When a molecular layer is inserted at this stage, it can modify adhesion, wettability, and the structural continuity of the overlying transport layer, which in turn changes the initial conditions for subsequent perovskite growth. Li and co-workers showed more directly how a molecular layer can reshape the buried hole-contact before perovskite deposition [77]. As illustrated in Fig. 4d, 2PACz was introduced between NiO_x_ and PVK, where the phosphonic-acid group anchors to the oxide surface and promotes a better-defined organic/inorganic interface. The interfacial electronic response also changed markedly: The transient charge signal and the corresponding electrostatic potential profile indicated a distinct interfacial dipole after 2PACz insertion (Fig. 4e). More importantly, this interfacial reconstruction was reflected in the emissive quality of the overlying layer. Whereas PVK on bare NiO_x_ showed a PLQY of only 13.9%, the NiO_x_/2PACz/PVK stack reached 85.0%, approaching the 91.0% measured on quartz and far exceeding bare NiO_x_ (6.5%) (Fig. 4f). These results indicate that introducing 2PACz does not simply modify the final contact energetics, but markedly improves the local interfacial environment beneath the hole-transport layer and suppresses interfacial quenching of the overlying perovskite emission. Yu and co-workers further showed that the effect of phosphonic-acid-based carbazole SAMs can already be seen at the level of buried-contact integrity [93]. Compared with PVK directly deposited on ITO, the introduction of 4PACz and Me-4PACz increased the mechanical robustness of the overlying PVK layer, as reflected in the larger pull-off force and higher adhesive energy, with Me-4PACz/PVK giving the strongest interfacial adhesion (Figs. 4g, h). Kelvin probe measurements further showed a clear difference in contact potential between ITO/4PACz and ITO/Me-4PACz (Fig. 4i), indicating that the more compactly packed monolayer also altered the local surface electronic environment. Taken together, these results suggest that improved SAM packing does not simply strengthen monolayer formation itself, but helps define a more intact and better-organized buried contact for the layers deposited above it.

**Fig. 5:**
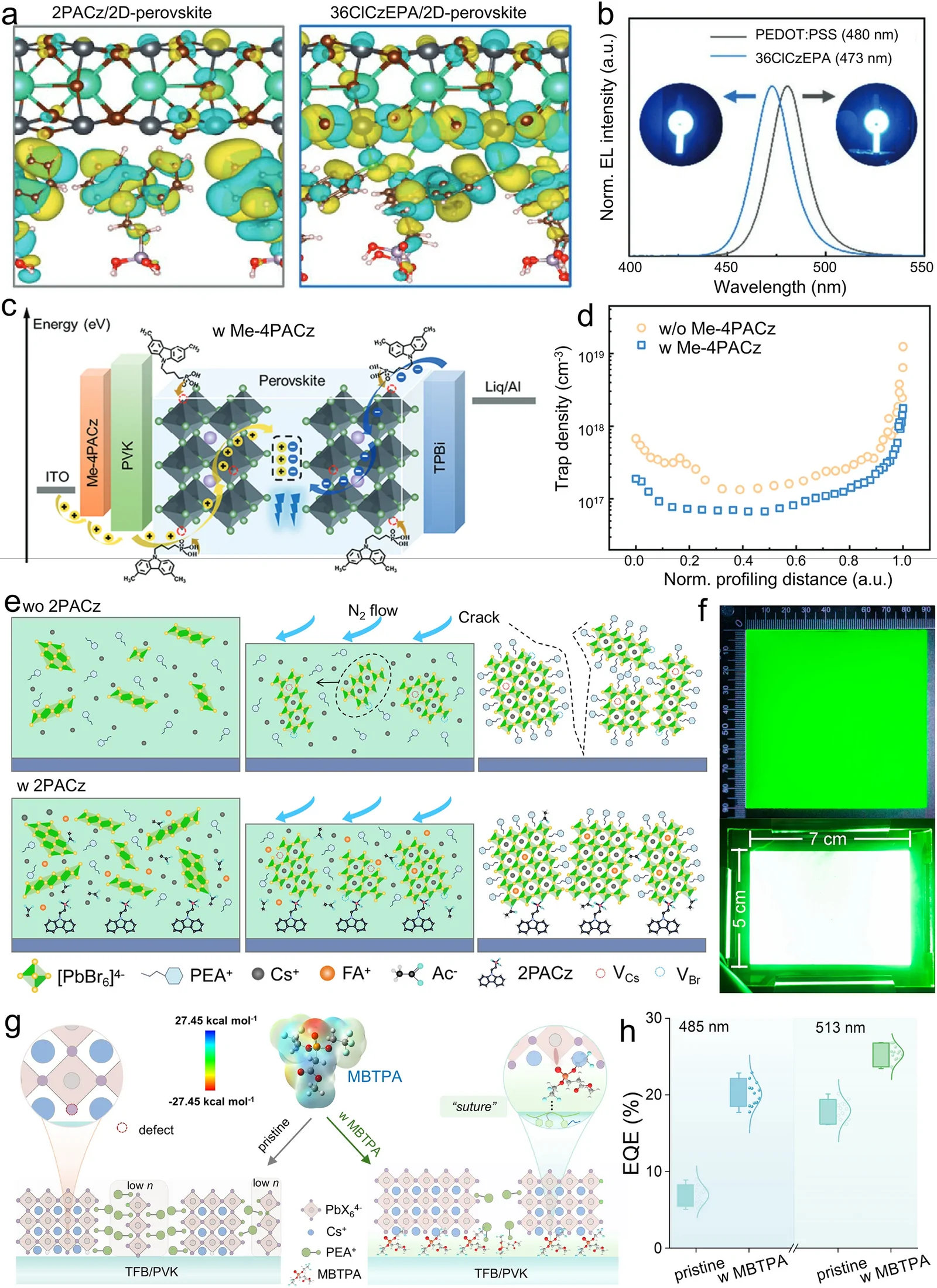
**a** Charge-density distribution at the buried interface for 2PACz/2D-perovskite and 36ClCzEPA/2D-perovskite configurations.** b** Normalized electroluminescence spectra of devices with PEDOT:PSS and 36ClCzEPA [89]. Copyright 2022, Wiley-VCH. **c** Schematic illustration of Me-4PACz-modified hole-contact interface. **d** Spatially resolved trap-density profiles for devices with and without Me-4PACz [79]. Copyright 2024, Wiley-VCH. **e** Schematic illustration of blade-coated perovskite film formation with and without 2PACz.** f** Large-area photoluminescence and electroluminescence images of blade-coated devices [10]. Copyright 2025, Elsevier.** g** Schematic illustration of the molecular-suturing strategy using MBTPA at the buried interface. **h** Statistical EQE distributions of blue (485 nm) and green (513 nm) devices with and without MBTPA [88]. Copyright 2026, Wiley-VCH

Once the interfacial layer defines a more intact and better-organized bottom contact, it also changes the environment in which the overlying perovskite layer forms. In other words, the molecular layer can affect not only interfacial energetics, but also nucleation, crystal growth, and the structural quality of the emissive film deposited above it. In pure-blue quasi-2D devices, Shin and co-workers showed that replacing 2PACz with (2-(3,6-dichloro-9H-carbazol-9-yl)ethyl) phosphonic acid (36ClCzEPA) changed not only the interfacial energetics, but also the local structural environment of the adjacent perovskite[89]. The calculated charge-density distribution indicated stronger interfacial electronic coupling in the 36ClCzEPA/2D-perovskite configuration than in the 2PACz-based counterpart (Fig. 5a). This difference was accompanied by a blue shift of the electroluminescence peak from 480 to 473 nm (Fig. 5b), consistent with a change in phase organization and local emissive environment near the bottom interface. Relatedly, Yu and co-workers showed that the bottom microstructure-induced capillary flow can also reshape the local crystallization environment, producing highly oriented ultrathin monolayer quasi-2D perovskites with suppressed trapping and ion migration [94]. This structural advantage is reflected in the improved spectral and device characteristics, enabling pure-blue electroluminescence at 466 nm with CIE coordinates of (0.132, 0.065), together with a peak EQE of 15.0% and stable EL spectra during operation. Li and co-workers showed that introducing Me-4PACz at the hole-contact side not only adjusted interfacial energetics, but also modified the local growth environment of the perovskite [79]. As illustrated in Fig. 5c, the SAM-assisted interface promotes more uniform carrier distribution and suppresses defect-related pathways near the contact. This change is reflected in the spatially resolved trap-density profile, where the Me-4PACz-modified device exhibits a consistently lower trap density across the film compared with the control (Fig. 5d). These results suggest that the buried molecular layer influences how the perovskite layer forms and stabilizes near the interface, rather than acting only on the completed device.

In blade-coated large-area CsPbBr_3_ PeLEDs, Chen and co-workers showed that introducing 2PACz at the buried interface changed the nucleation and crystallization pathway during film formation [10]. Without 2PACz, the growing film developed discontinuous crystal domains and was more prone to cracking during solvent removal; with 2PACz, nucleation became more uniform and the resulting crystal domains were better connected, yielding a more continuous emissive layer (Fig. 5e). This difference was reflected at the device scale: The blade-coated films showed uniform large-area photoluminescence, and the corresponding 5 cm × 7 cm devices exhibited spatially continuous electroluminescence (Fig. 5f). This coupling between interfacial chemistry and film growth is further highlighted by a recent “molecular suturing” strategy reported by Cao and co-workers [88]. Although methyl bis(2,2,2-trifluoroethyl) phosphonoacetate (MBTPA) is not presented as a canonical SAM, it functions in a closely related way as an ultrathin buried interfacial molecule. As shown in Fig. 5g, MBTPA simultaneously strengthens the HIL/perovskite contact and affects crystal growth at the bottom interface. In this design, the C=O and P=O groups interact with undercoordinated Pb^2+^ and halide-vacancy-related defects, whereas the -CF_3_ group forms hydrogen-bonding interactions with the hole-injection layer, thereby coupling interfacial adhesion, defect passivation, and bottom-interface crystallization. The corresponding devices reached peak EQEs of 23.14% for blue emission and 27.05% for green emission (Fig. 5h), further showing that ultrathin interfacial molecules can shape both the contact itself and the way the emissive film develops above it. In co-evaporated perovskite systems, Feeney and co-workers found that phosphonic-acid groups exposed at the substrate can coordinate with halide species during deposition, thereby modifying precursor adsorption and increasing nucleation density at the interface [95]. This leads to a more uniform initial growth front and suppresses the formation of interfacial defects. Castro-Méndez et al. demonstrated that phosphonic acids can tune interfacial energetics and stabilize the cubic *α*-FAPbI_3_ phase during room-temperature evaporation, inhibiting phase segregation [96]. In this sense, interfacial molecular chemistry affects not only energy-level alignment, but also the selection of crystal phase and the defect landscape established during film growth.

The picture that emerges is therefore more specific than the generic statement that “SAMs improve interfaces.” In PeLEDs, SAMs can act as molecularly defined bottom-interface templates that first determine how the buried contact is assembled, then influence how the emissive layer nucleates or organizes above it, and finally manifest as changes in radiative efficiency and device operation. This coupled structural–electronic role is likely to be most consequential in quasi-2D, nanocrystalline, and blue-emitting systems, where small variations in bottom-interface organization readily translate into large differences in phase distribution, carrier confinement, and non-radiative loss.

### Local Electronic-Type Modulation of Perovskites

Beyond their effects on bottom-interface formation and perovskite crystallization, interfacial molecules can also modify the local electronic character of the adjacent perovskite region. In halide perovskites, this near-interface electronic state is sensitive to charge transfer, molecular dipoles, and defect redistribution. Molecular layers can therefore shift the local Fermi-level position, alter carrier polarity, and reshape the carrier population near the contact, in some cases producing p- or n-enriched regions or local band bending. Such effects are directly relevant to PeLEDs because they influence injection selectivity, recombination-zone location, and interfacial quenching.

**Fig. 6:**
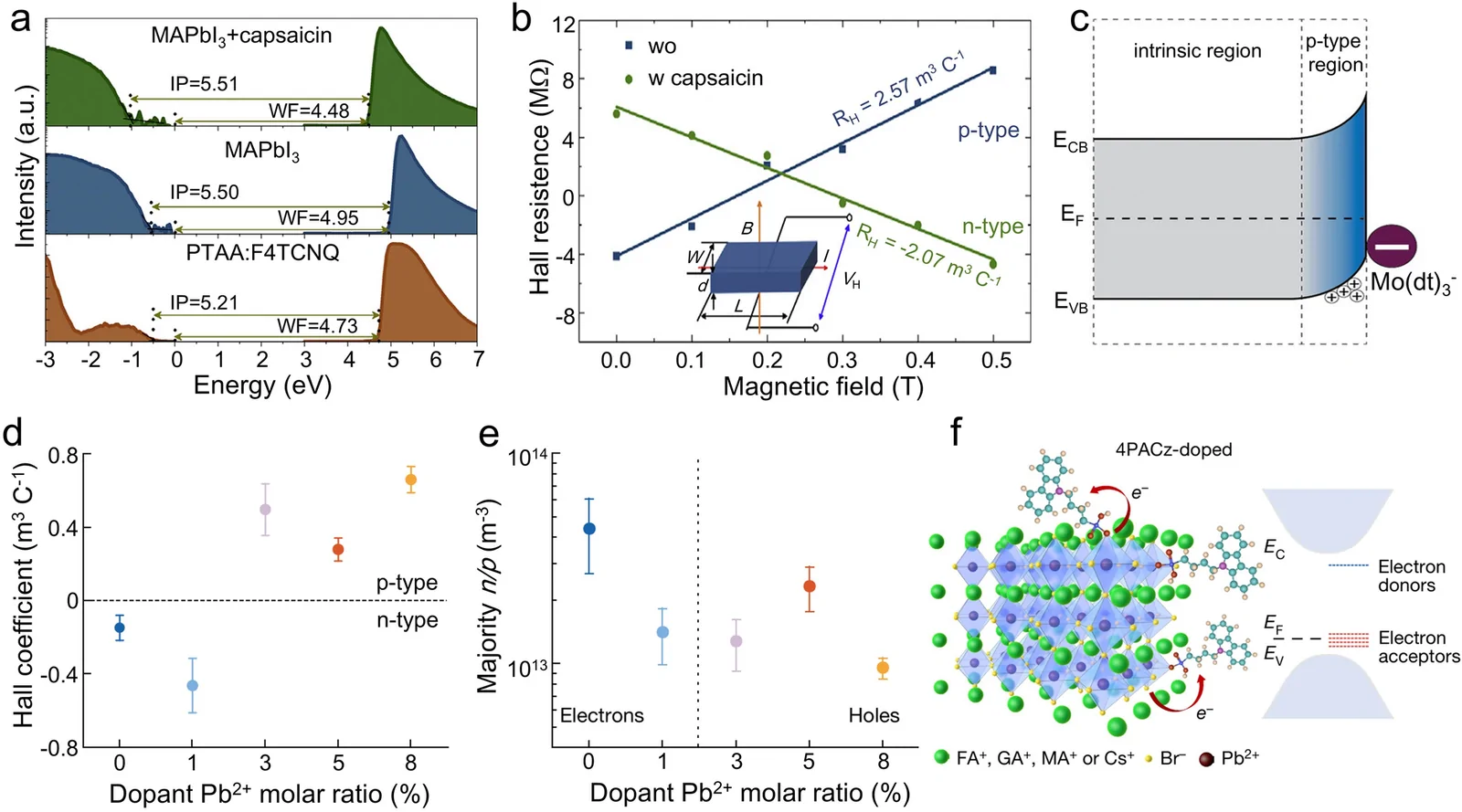
**a** Ultraviolet photoelectron spectroscopy (UPS) spectra showing the shift in work function (WF) of MAPbI_3_ after capsaicin treatment.** b** Hall effect measurement of perovskite films. The insert shows Hall effect measurement setup [97]. Copyright 2021, Elsevier.** c** Illustration of proposed band bending at the surface of the perovskite crystal due to the charge-transfer reaction with Mo(tfd-CO_2_Me)_3_ [98]. Copyright 2019, Royal Society of Chemistry. **d** Evolution of the Hall coefficient as a function of the Pb^2+^ molar ratio of the 4PACz dopant.** e** Majority carrier concentration as a function of 4PACz doping level. **f** Schematic illustration of p-doping in perovskites induced by 4PACz, together with energy-level diagrams comparing the doped systems [9]. Copyright 2024, Nature Publishing Group

The local electronic effect of interfacial molecules has also been observed directly. Xiong and co-workers showed that capsaicin treatment changed the near-surface electronic character of MAPbI_3_ [97]. The work function shifted from 4.95 to 4.48 eV while the ionization potential remained almost unchanged, consistent with a substantial movement of the near-surface Fermi level (Fig.6a). Hall measurements then showed a transition from p-type to n-type behavior (Fig. 6b), and the resulting p-n homojunction was located about 100 nm below the surface. This result indicates that molecular treatment can modify not only defect density, but also the local polarity of the perovskite close to the interface. A related situation was reported by Noel et al., where molecular treatment induces p-type character through interfacial charge transfer [98]. The Mo(dt)_3_ dopant extracts electrons from the perovskite surface, shifting the Fermi level toward the valence band and generating a hole-enriched region with upward band bending near the interface (Fig. 6c). Jiang et al. showed that remote molecular treatment can create an n-type low-dimensional surface layer on a wide-bandgap 3D perovskite [99]. This treatment produced a surface heterojunction with the underlying film. The modified region showed n-type behavior, carrier densities on the order of 10^16^ cm^−3^, and a Fermi level shifted toward the conduction band. Zhai and co-workers reported a related effect for self-assembled molecules on perovskite top surfaces, where large molecular dipoles were used to tune interfacial energetics and reduce energetic offsets without requiring a uniform change in the bulk conductivity type [100]. They showed that large molecular dipoles can tune interfacial energetics and reduce energetic offsets. These studies matter because they suggest that many molecule-induced “electronic-type” effects in perovskites first appear as local carrier enrichment, Fermi-level displacement, or dipole-driven band bending, rather than as a uniform bulk polarity conversion.

In 2024, Xiong and co-workers reported an emissive perovskite in which the electronic polarity could be tuned in a controlled manner [9]. Using a phosphonic-acid molecular (4PACz) dopant with strong electron-withdrawing ability, they tuned a wide-bandgap emissive perovskite between n-type and p-type behavior. As shown in Fig. 6d, the Hall coefficient changed systematically with dopant concentration, crossing from − 0.5 to 0.6 m^3^ C^−1^. The majority-carrier density also shifted from electron- to hole-dominated regimes and remained above 10^13^ cm^−3^ (Fig. 6e). The proposed mechanism is illustrated in Fig. 6f: The molecular dopant acts as an electron acceptor and modifies the local electronic balance of the perovskite through charge transfer. Importantly, this polarity control was achieved while retaining high PLQYs of 70%–85%. The resulting HTL-free PeLEDs delivered more than 1.1 × 10^6^ cd m^−2^ brightness and an EQE of 28.4%. This study demonstrated that electronic-type control in perovskites is compatible with high emissive quality. Qin et al. reported that additive engineering can enhance the hole concentration at the perovskite surface [101]. They linked this local hole enrichment to improved luminescence yield and more efficient electroluminescence at low current density.

Local electronic-type modulation is therefore better understood as part of a continuum of interfacial regulation rather than as an isolated phenomenon. Depending on the molecular system and the perovskite composition, the observable outcome may appear as Fermi-level displacement, local carrier enrichment, dipole-induced band bending, or, in some cases, a more explicit change in near-interface carrier polarity. For PeLEDs, these effects are relevant not because they represent a separate category of interface engineering, but because they can alter charge selectivity at the contact, reshape the local recombination environment, and change how strongly interfacial quenching develops under bias. In this respect, the discussion by Zou and co-workers is instructive: Lewis basic molecules in PeLEDs were considered not only in terms of defect passivation, but also in terms of their coupled influence on crystallization, defect chemistry, and interfacial electronic processes [102]. Molecular regulation of the near-interface electronic state is closely intertwined with the structural and chemical roles of the same interfacial layer, and is most meaningfully considered together with them in analyzing PeLED operation.

### Top-Interface Contacts and Interfacial Loss

The discussion above shows that buried-interface molecules regulate not only initial contact energetics, but also the nucleation, adhesion, and structural stabilization of the emissive layer near the substrate. Under electrical driving, the perovskite/electron-transport-layer (ETL) junction becomes another critical region, because electron injection, exciton confinement, interfacial quenching, and halide redistribution converge at the cathode side. The top interface in PeLEDs should therefore be considered not simply as an electron-injection boundary, but as a contact where excited-state loss, interfacial compatibility, and bias-dependent stability are closely coupled.

**Fig. 7:**
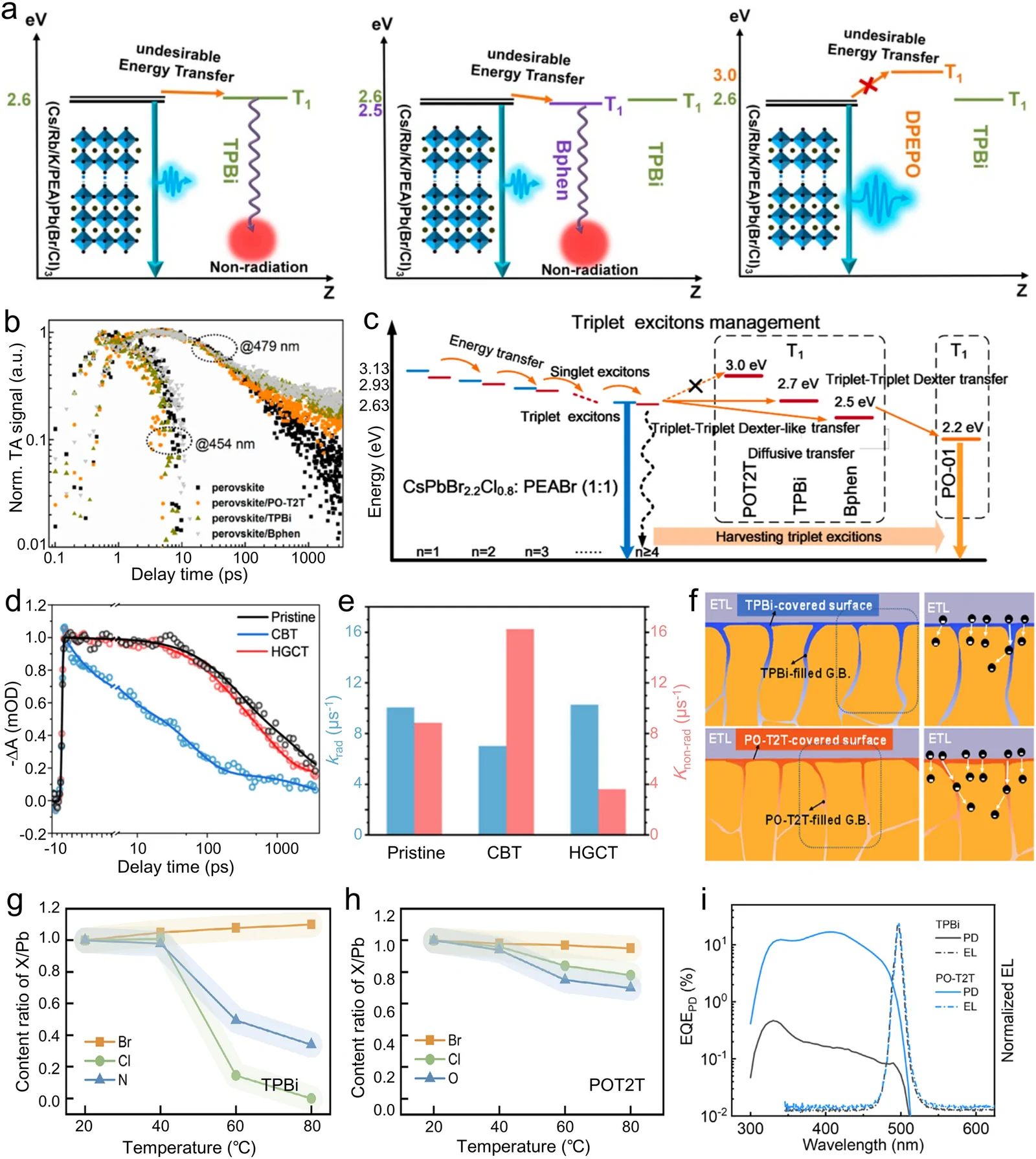
**a** Schematic illustration of undesirable interfacial energy transfer from sky-blue perovskites to TPBi or Bphen, and its suppression by inserting DPEPO with a higher triplet energy level at the perovskite/ETL interface. Copyright 2021, American Chemical Society.** b** Normalized transient-absorption dynamics of quasi-2D perovskite films and perovskite/organic-contact bilayers. **c** Energy-level diagram for triplet-exciton management at the top interface, illustrating triplet–triplet Dexter-like transfer from quasi-2D perovskites to an ultrathin PO-01 layer through different ETL spacers (PO-T2T, TPBi, and Bphen). Copyright 2022, American Chemical Society.** d** Transient-absorption decay kinetics of pristine, CBT, and HGCT samples in all-solution-processed sky-blue systems. **e** Extracted radiative (*k*_rad_) and non-radiative (*k*_non-rad_) rate constants. Copyright 2026, American Chemical Society.** f** Schematic comparison of TPBi- and PO-T2T-modified cathode-side contacts. Copyright 2023, American Chemical Society. **g, h** Temperature-dependent compositional evolution at the perovskite/TPBi and perovskite/PO-T2T interfaces, respectively. Copyright 2026, Wiley-VCH.** i** Wavelength-dependent photodetection EQE_PD_ and normalized EL spectra of bifunctional perovskite diodes using TPBi or PO-T2T. Copyright 2026, Wiley-VCH

A recurring issue at the upper interface is exciton loss, especially when commonly used organic ETLs directly contact the perovskite surface. As illustrated in Fig. 7a, direct contact of the sky-blue perovskite with TPBi or bathophenanthroline (Bphen) creates an unfavorable upper-interface energy-transfer pathway, because the T_1_ levels of TPBi (2.6 eV) and Bphen (2.5 eV) lie below that of the perovskite and can therefore accept interfacial excitations, leading to non-radiative loss [103]. By contrast, inserting DPEPO with a higher *T*_1_ level (3.0 eV) blocks this undesirable transfer channel while preserving the electron-transport function of the cathode-side contact. Under this configuration, Zhu and co-workers suppressed interfacial excited-state loss at the perovskite/ETL junction and achieved a maximum luminance of 55,000 cd m^−2^. This comparison shows more directly that the perovskite/ETL contact controls not only electron injection, but also whether excitations formed near the upper interface are quenched or retained for radiative recombination. A related conclusion was reached by Yu and co-workers in quasi-2D perovskites [104]. As reflected in Fig. 7b, the normalized transient-absorption dynamics at 479 and 454 nm differ markedly after introducing different cathode-side contacts, indicating that the upper interface strongly affects excited-state evolution in the quasi-2D emissive layer. The corresponding energy-level analysis in Fig. 7c further shows that the T₁ levels of PO-T2T (3.0 eV), TPBi (2.7 eV), and Bphen (2.5 eV) lie within the range relevant to interfacial triplet management, while the ultrathin orange phosphorescent layer bis(4-phenylth-ieno[3,2-*c*]pyridine) (acetylacetonate) iridium(III) (PO-01) (2.2 eV) provides an energetically accessible acceptor for triplet–triplet Dexter-like transfer. Under this design, triplet excitons generated in the quasi-2D perovskite are no longer lost predominantly at the upper interface, but can be transferred and harvested through the cathode-side organic layer. The resulting white devices reached a maximum forward-viewing EQE of 8.6% and a luminance above 15,000 cd m^−2^, compared with 4.6% for the corresponding sky-blue perovskite device, showing that the perovskite/ETL contact can be tuned from an interfacial loss channel into a functional excited-state management interface.

A more process-oriented example was reported by Jiang and co-workers in all-solution-processed sky-blue devices [105]. Through an integrated supramolecular host-guest post-treatment strategy, they reconstructed upper-contact compatibility and achieved an EQE of 14.75%, a maximum luminance of 8740 cd m^−2^, and stable sky-blue electroluminescence centered at 495 nm with a narrow 15.8 nm linewidth. In the transient-absorption traces (Fig. 7d), the CB-treated (CBT) sample exhibits the fastest decay, whereas the host-guest complex-treated (HGCT) sample decays more slowly and maintains a much stronger long-lived signal, indicating suppressed interfacial loss after upper-contact reconstruction. The extracted rate constants further show that CBT is dominated by enhanced non-radiative decay, with *k*_non-rad_ markedly increased and *k*_rad_ reduced, while HGCT lowers *k*_non-rad_ to the smallest value among the three cases and restores *k*_rad_ to a level comparable to or slightly higher than the pristine film (Fig. 7e). These results show that the cathode-side interface affects not only contact formation under solution processing, but also the radiative versus non-radiative balance of the emissive layer after ETL formation. Beyond exciton loss, the upper contact can also directly regulate charge behavior through interfacial bonding and contact formation. Zhao and co-workers showed that replacing the conventional TPBi contact with PO-T2T rebuilt the EML/ETL junction into a chemically bonded interface and enabled a maximum EQE of 22.06% together with a maximum luminance of 103,286 cd m^−2^ [106]. As illustrated in Fig. 7f, the difference is not only energetic but also morphological: TPBi mainly forms a surface-covered contact, whereas PO-T2T covers the perovskite surface while also filling grain boundaries, thereby creating a more continuous cathode-side junction. Under this configuration, the upper interface is no longer a simple extraction boundary, but a contact that reshapes local charge distribution near the perovskite/ETL region and suppresses interfacial loss during device operation. These results therefore place the perovskite/ETL junction directly in the performance-determining pathway, where contact chemistry, interfacial coverage, and regulated charge behavior act together.

The same interface also becomes critical once thermal stress is introduced. Li and co-workers recently showed that thermal degradation in mixed-halide blue PeLEDs is strongly ETL-dependent [107]. Devices using TPBi exhibited rapid failure because of weak physical interfacial contact, thermal detachment, and severe morphological change, whereas PO-T2T-based devices retained about 90% of their initial efficiency at 80 °C owing to stronger chemical bonding and higher thermal stability at the interface. As shown in Figs. 7g, h, this difference is directly reflected in the interfacial compositional evolution. In TPBi-based devices, the Cl/Pb ratio decreases sharply from about 1 at 20–40 °C to nearly 0 at 80 °C, accompanied by a pronounced drop in the N/Pb ratio to about 0.35, indicating severe halide loss and ETL detachment at the cathode-side contact. By contrast, PO-T2T-based devices retain much higher interfacial compositional integrity, with Br/Pb remaining close to unity and both Cl/Pb and O/Pb still around 0.7–0.8 at 80 °C. In situ photoelectron spectroscopy further showed migration of halides from the perovskite into both ETLs, linking EL spectral shift under heating to top-contact evolution and interfacial chemical instability.

This top-interface logic can also be seen in recent bifunctional perovskite diodes. Zhang and co-workers realized stable dual-mode operation by combining improved emissive-phase control with a dissociative type-II perovskite/ETL heterojunction at the cathode side [108]. As shown in Fig. 7i, changing the ETL from TPBi to PO-T2T leaves the EL peak essentially unchanged near 500 nm, but strongly reshapes the photodetection response: The EQE_PD_ increases from only 0.46% for the TPBi-based device to 16.62% for the PO-T2T-based device, that is, by about 36 times. This contrast indicates that the upper heterojunction can be designed to preserve efficient electroluminescence while simultaneously promoting exciton dissociation and carrier separation under reverse bias. In this sense, the perovskite/ETL contact is not a passive boundary, but a tunable cathode-side junction whose electronic character determines whether the interface favors exciton-preserving emission or carrier-separating operation.

These studies define a more specific role for the top interface in PeLEDs. The buried side largely determines how the emissive layer forms and how the adjacent perovskite region is electronically conditioned, the upper contact determines how that layer behaves once the ETL is introduced and the device is driven. Its influence is expressed through interfacial excited-state loss, charge redistribution, contact compatibility, halide retention, and thermal stability. A balanced discussion of molecular interface engineering in PeLEDs therefore needs to treat the perovskite/ETL junction as a major operating interface, rather than as a secondary electron-injection boundary considered only after buried-interface optimization.

### Device-Level Manifestations of Molecular Modified Interfaces

Once molecules were introduced into PeLEDs as practical interfacial layers, their effects began to be reflected directly in device operation. What changes first is not the device metric itself, but the interfacial processes that determine how carriers are injected, accumulated, and recombined under bias. Accordingly, variations in turn-on voltage, transient response, EQE, efficiency roll-off, and operational stability can be read as device-level manifestations of changes in injection barriers, interfacial trapping, charge balance, and the persistence of local electric fields at working interfaces. This perspective is useful because it links molecular modification at the interface to experimentally observable device behavior without treating each metric as an isolated outcome.

**Fig. 8:**
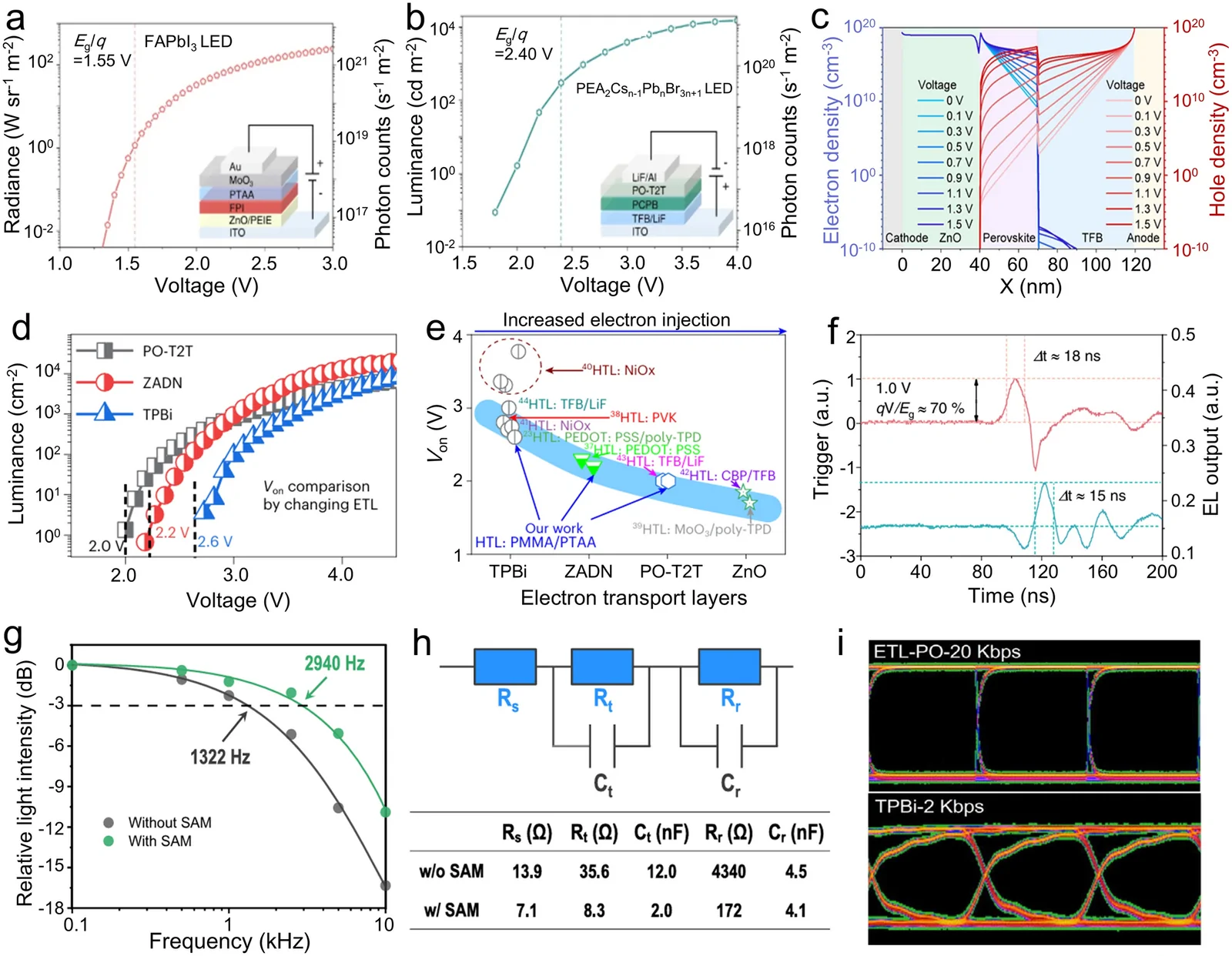
**a** Radiance-voltage characteristics of an FAPbI_3_ (FPI) PeLED with the corresponding device structure shown in the inset. **b** Luminance-voltage characteristics of a PEA_2_Cs_n-1_Pb_n_Br_3n+1_ (PCPB) PeLED with the corresponding device structure shown in the inset. **c** Simulated electron and hole density distributions across the device under different applied voltages [109]. Copyright 2022, Nature Publishing Group. **d** Luminance-voltage characteristics of devices using different electron-transport layers (PO-T2T, ZADN, and TPBi), with the corresponding turn-on voltages marked. **e** Comparison of turn-on voltages reported for devices with different electron-transport layers, arranged according to increasing electron injection [101]. Copyright 2025, Nature Publishing Group. **f** Electrical trigger pulse and electroluminescence output pulse recorded at 1.0 V, showing the input and output pulse widths [109]. Copyright 2022, Nature Publishing Group.** g** Frequency-dependent relative light intensity for devices with and without SAM modification, with the corresponding cutoff frequencies indicated. **h** Equivalent-circuit model used for impedance analysis, together with the fitted resistance and capacitance values for devices with and without SAM modification [77]. Copyright 2023, Nature Publishing Group. **i** Eye diagrams recorded for devices with different electron-transport interfaces, showing signal transmission at the indicated data rates [111]. Copyright 2026, Wiley-VCH

Turn-on voltage (*V*_on_) is one of the earliest device-level manifestations of interfacial regulation, because it depends on how readily electrons and holes can be injected from the two contacts and brought into spatial overlap under bias. Lian and co-workers showed that perovskite LEDs can emit at voltages well below the corresponding photon energy, with electroluminescence observed at 1.55 V for an iodide near-infrared device and 2.40 V for a bromide green device, as shown in Figs. 8a, b [109]. This behavior indicates that turn-on is not determined solely by a nominal bandgap-derived threshold, but also by how efficiently carriers are injected and accumulated at the working interfaces. The simulated carrier distributions show that as the bias increases, electron and hole populations build up near the opposite interfaces and progressively overlap within the perovskite layer, allowing radiative recombination to be established before the applied voltage reaches the nominal photon-energy limit (Fig. 8c). A related point was made by Zhang and co-workers, who used a 2PACz hole-injection monolayer on ITO and showed that the monolayer affected work-function tuning, hole injection, and radiative recombination at the bottom interface [110]. The electron-transport side also has a clear influence on turn-on behavior. Qin and co-workers showed that changing the ETL from TPBi to ZADN and then to PO-T2T progressively reduced *V*_on_ from 2.6 to 2.2 and 2.0 V, respectively (Fig. 8d) [101]. As summarized in Fig. 8e, this trend is consistent with more efficient electron injection. These results indicate that *V*_on_ is shaped by the injection conditions at both contacts, and that improved interfacial quality on either side can lower the voltage required to establish efficient electroluminescence.

The same interface dependence is reflected in transient response. If carrier injection and recombination can be established with lower loss and less interfacial charging, the kinetic penalty associated with switching is also reduced. Lian and co-workers showed that a PeLED operated at 1.0 V delivered an optical output pulse width of ~ 15 ns for an input trigger pulse of ~ 18 ns (Fig. 8f), indicating that fast electroluminescent switching can be maintained when carrier injection and recombination remain efficient at the working interfaces [109]. Using a NiO_x_/2PACz/PVK hole-contact architecture, Li and co-workers showed that SAM introduction reduced the total response time from 540 to 373 μs and increased the cutoff frequency from 1322 to 2940 Hz (Fig. 8g) [77]. The electrochemical impedance spectroscopy analysis in Fig. 8h further showed that SAM introduction reduced both the interfacial resistance and capacitance, consistent with more efficient charge transport across the hole-contact interface. A similar trend was reported by Ren and co-workers in CsPbBr_3_ perovskite quantum-dot LEDs, where optimization of the electron-transport interface improved both charge injection and carrier extraction [111]. The optimized device showed a total response time of 564 ns at an active area of 0.01 mm^2^, more than 50 times faster than the 29 μs response of the control device. This difference is also reflected in the eye diagrams in Fig. 8i: The ETL-optimized device maintained a clear open signal at 20 Kbps, whereas the TPBi-based control already showed pronounced distortion at 2 Kbps. These results indicate that transient response is highly sensitive to interfacial charging and discharge processes, and that improving interface quality can shift PeLED operation from the microsecond regime toward the nanosecond regime.

**Fig. 9:**
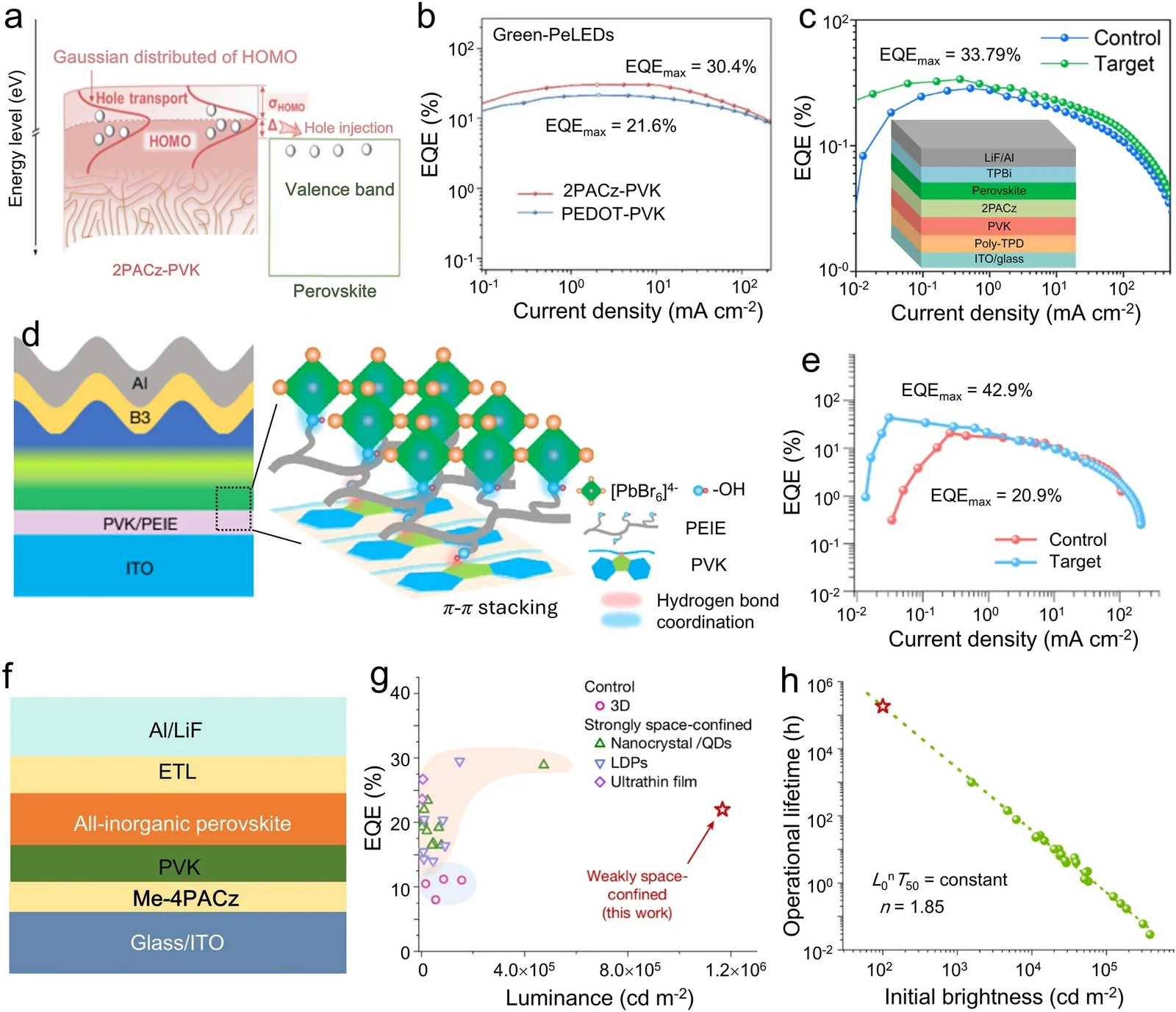
**a** Schematic illustration of energetic disorder at the hole-contact interface and its reduction by 2PACz modification, leading to a more favorable hole-injection landscape in the PVK layer. **b** Current density-dependent EQE of green PeLEDs comparing 2PACz-modified and PEDOT:PSS-based hole contacts [76]. Copyright 2025, American Chemical Society. **c** EQE-current density characteristics of a 2PACz-based device and control, with the optimized device reaching a peak EQE of 33.79% [75]. Copyright 2026, Wiley-VCH. **d** Schematic of enhanced molecular packing at the hole-contact interface via π-π stacking and interfacial coordination, leading to a more ordered interfacial structure. **e** Corresponding EQE characteristics showing an increase in peak EQE from 20.9% to 42.9% [16]. Copyright 2026, Nature Publishing Group. **f** Device architecture of all-inorganic PeLED using Me-4PACz at the hole-contact interface. **g** Comparison of EQE-luminance performance for different confinement regimes. **h** Half-lifetime versus brightness from accelerated lifetime tests of weakly space-confined PeLEDs [54]. Copyright 2025, Nature Publishing Group

Under sustained operation, the same interface conditions continue to influence steady-state device behavior. When non-radiative loss near the contact is reduced and charge balance is improved, the first consequence is usually an increase in peak EQE. As the current density rises, the same interfaces determine whether carriers remain balanced or begin to accumulate locally, which is then reflected in the extent of roll-off. During longer operation, these differences further shape how quickly degradation develops under sustained bias. Over longer operation times, these same interfacial conditions further control whether the device can maintain a stable recombination zone, suppress interfacial quenching, and resist thermally or electrically induced chemical evolution. Luo and co-workers showed that a 2PACz-modified hole-contact reduced energetic disorder in the overlying PVK layer (Fig. 9a) [76]. As a result, the device reached a peak EQE of 30.4% (Fig. 9b) and exhibited a ninefold increase in operational lifetime. In blue devices, the same strategy increased the EQE from 15.5% to 25.3%. In a related study based on 2PACz, Chen and co-workers showed that improving the local film-formation environment at the hole-contact interface could also be reflected directly in device performance [75]. In their 2PACz-based architecture, the target device reached a peak EQE of 33.79%, higher than that of the control, while maintaining improved reproducibility, as shown by the EQE-current density characteristics in Fig. 9c. Yu and co-workers showed that improving the molecular packing of phosphonic-acid-based carbazole SAMs enabled the formation of more intact monolayers, which in turn reduced interfacial defect-assisted non-radiative recombination and produced an approximately 30% enhancement in PeLED efficiency [93]. A further example highlights how interfacial organization can translate into device efficiency. In a recent study, molecular packing at the hole-contact interface was strengthened through π-π stacking (Fig. 9d), leading to a more ordered interfacial structure. The corresponding device showed a peak EQE increase from 20.9% to 42.9% (Fig. 9e), indicating that improved interfacial ordering can substantially enhance radiative recombination efficiency [16]. Peng et al. further showed that weakly space-confined green PeLEDs can retain high efficiency under much more demanding operating conditions [54]. In the device structure shown in Fig. 9f, the EQE remained above 20% at current densities near 1000 mA cm⁻^2^. The same devices also combined high efficiency with high brightness, reaching a region that extends beyond the performance range of more strongly space-confined systems (Fig. 9g). In addition, the extrapolated half-lifetime reached 185,600 h at 100 cd m^−2^, as summarized in Fig. 9h.

**Fig. 10:**
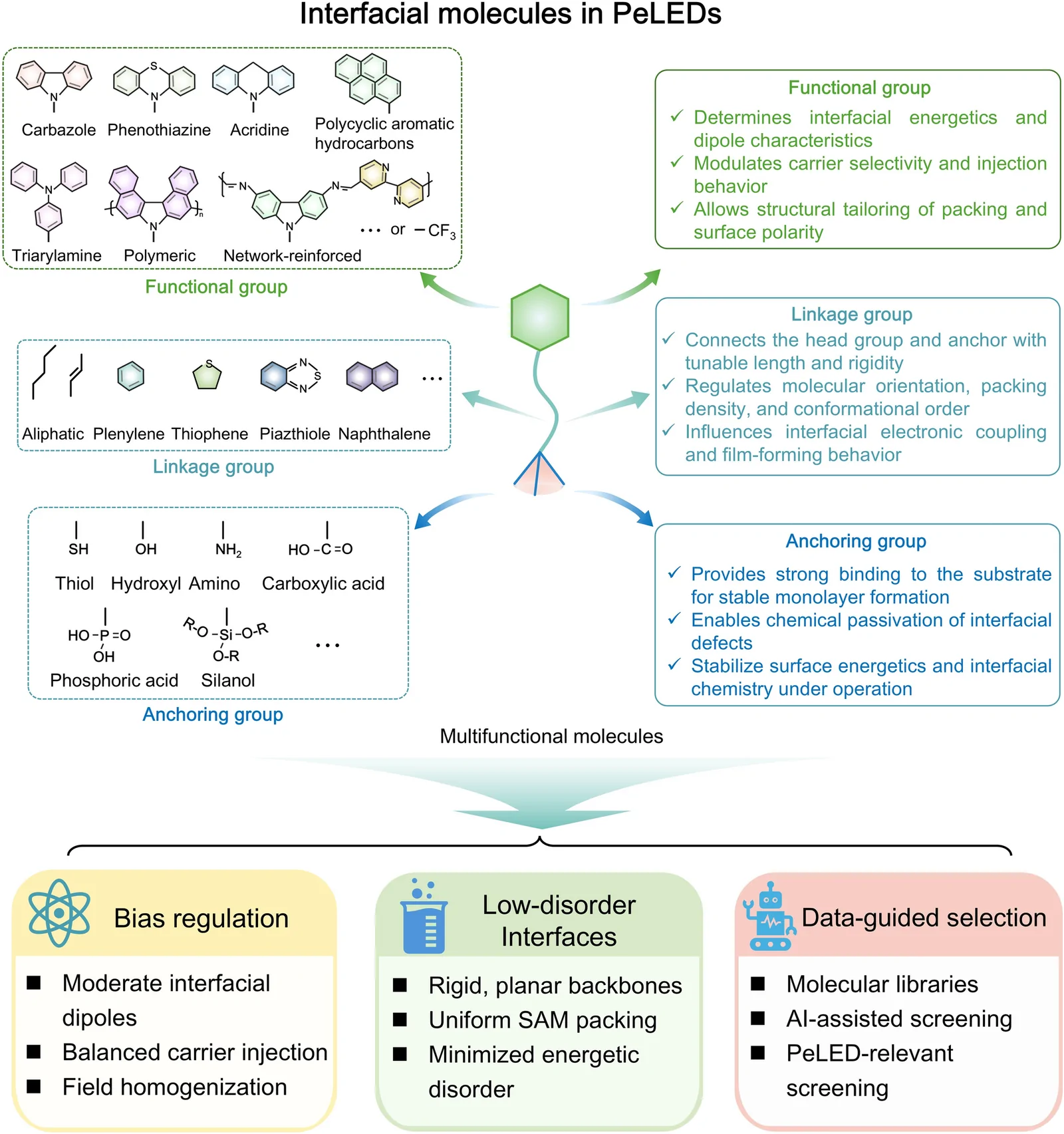
Conceptual framework linking molecular composition to interfacial functions and design considerations in PeLEDs

The stability trends collected in Table 1 further show that molecular interface design can influence device lifetime over a very broad range, from only several minutes to hundreds or even more than ten thousand hours, depending on the molecule, device architecture, and testing condition. For example, representative 2PACz-based devices show *T*_50_ values ranging from 75 h at 100 cd m^−2^ to 148.4 min and 16 min at 100 cd m^−2^ in different structures, indicating that the same molecular motif can lead to very different stability outcomes depending on the surrounding transport layers and emissive system. More pronounced lifetime improvements are observed in some optimized systems, such as I-2PACz with 11,852 h at 100 cd m^−2^, Br-2PACz with 350 h at 1000 cd m^−2^, MeO-2PACz with 14.5 h at 1000 cd m^−2^, 2BrCzPA with 360 h at 1000 cd m^−2^, and Me-4PACz with an extrapolated half-lifetime of 185,600 h at 100 cd m^−2^. These comparisons suggest that interfacial molecules do not improve stability through a single mechanism; rather, their effects depend on how well they simultaneously support charge balance, defect passivation, crystallization control, interfacial adhesion, and chemical robustness under bias (Fig. 10).

These studies show that the effects of interfacial molecular regulation in PeLEDs are reflected across a connected set of operating characteristics rather than in a single device metric. Once the interfaces are altered, the consequences appear first in carrier injection and overlap, then in switching and high-current operation, and finally in long-term stability. This progression also makes clear why the question of molecular selection in PeLEDs cannot be separated from device operation itself.

## Rational Selection and Design of Interfacial Molecules for PeLEDs

The preceding sections suggest that molecular selection in PeLEDs is better considered from the operating conditions of the device than from any single interfacial parameter. In a working PeLED, the emissive layer is thin, the carrier population changes over a wide range with current density, and both charge-injection interfaces can influence charge accumulation, non-radiative loss, and recombination-zone stability [23, 32, 89]. Under such conditions, the question is not simply whether a molecule lowers a nominal barrier, but whether the interface it creates remains compatible with electroluminescent operation over time. This is also consistent with recent discussions of Lewis basic molecules in PeLEDs, where the relevant functions are not limited to passivation or energy-level adjustment alone, but extend to precursor chemistry, crystallization, defect distribution, and interfacial electronic processes.

*Electronic regulation under electrical bias* For light-emitting devices, interfacial regulation needs to be judged under bias rather than only from static energy-level diagrams. A molecule that produces a large work-function shift may appear favorable in isolation, yet still become problematic if it drives charge accumulation near one contact or destabilizes the recombination zone at higher current density [77, 109]. In this sense, the more useful electronic effect is often moderate and spatially uniform. Dipole magnitude matters, but so do dipole orientation, packing, and the extent to which the interfacial field is distributed across the contact. The examples discussed in Sect. 2 indicate that changes in turn-on behavior, transient response, roll-off, and stability are often linked not to nominal barrier lowering alone, but to how effectively the interface regulates carrier density and charge balance during operation. This consideration also helps explain why molecular strategies that perform well in PSCs are not always directly transferrable to PeLEDs, where the relevant limitation is frequently recombination under injection rather than carrier extraction.

*Chemical persistence and structural compatibility* Electronic regulation cannot be separated from how the interface is formed and how it evolves under electrical stress. In PeLEDs, the molecular layer is already present before deposition of the overlying transport or emissive film, so it becomes part of the local environment that governs adhesion, wetting, nucleation, and defect formation [10, 88, 112]. The same layer must then remain intact during operation, when the interface is exposed to local heating, charge accumulation, and, in some systems, ion redistribution. This places combined demands on anchoring chemistry, monolayer integrity, chemical compatibility with adjacent layers, and resistance to solvent or thermal disturbance [79]. Because SAM performance depends strongly on substrate-specific chemisorption and packing, a molecule that forms a compact and well-oriented layer on one oxide surface may show incomplete coverage or less uniform organization on another substrate. Similarly, although ultrathin monolayers are attractive for minimizing optical and transport penalties, insufficient coverage can weaken carrier selectivity and electron-blocking capability, while unfavorable surface energy may complicate the deposition of subsequent transport or perovskite layers. These issues become more critical when moving beyond well-behaved oxide contacts to non-oxide, organic, or chemically complex interfaces. A molecule that improves bottom-interface ordering may also influence crystallization, and the resulting structural differences may later appear in recombination behavior and device stability. For PeLEDs, this coupled structural–electronic role is particularly important in quasi-2D, mixed-halide, nanocrystalline, and blue-emitting systems, where local disorder near the buried interface is readily reflected in phase distribution, confinement, and non-radiative loss.

*Molecular libraries, screening logic, and data-driven selection* Within this context, molecular selection is more informative when approached as a multidimensional screening problem than as the optimization of a single descriptor. The anchoring group affects chemisorption strength and processing tolerance. The linker influences molecular order and conformational freedom. The terminal functionality can alter dipolar regulation, precursor interaction, wettability, passivation strength, and, in some cases, exciton quenching [102]. These features are best evaluated together with device-relevant readouts, including turn-on behavior, transient electroluminescence, current-dependent EQE roll-off, high-brightness operation, and the reproducibility of the resulting film. A useful next step may therefore lie in building more systematic molecular libraries in which structural descriptors, interfacial measurements, and device outputs are recorded in a comparable way [113–115]. On that basis, machine learning and other data-driven approaches may become useful not simply for ranking molecules, but for identifying non-obvious combinations of anchoring, dipolar, and structural features that are compatible with PeLED operation. In the present context, such approaches would be most meaningful when they remain tied to experimentally accessible interfacial observables rather than abstract fitting variables alone.

## Conclusion and Outlook

This review has considered interfacial molecular regulation in PeLEDs from the perspective of carrier behavior, interface-limited device operation, and molecular design. In PeLEDs, interfaces are not simply internal boundaries between functional layers. They participate directly in charge injection, local carrier accumulation, non-radiative loss, recombination-zone formation, and the structural evolution of the emissive layer above the contact. For this reason, the role of interfacial molecules cannot be understood only through static energy-level alignment or defect passivation. Their influence often extends from bottom-interface formation and perovskite crystallization to local electronic regulation and, ultimately, to device operation under bias. Seen from this progression, interfacial molecules in PeLEDs are better viewed as coupled regulators of structure, chemistry, and electronics. The same molecular layer may affect precursor coordination, wetting, nucleation, defect distribution, charge selectivity, and interfacial stability at the same time. This also explains why similar molecular motifs can lead to different outcomes once incorporated into different emitters, transport layers, or processing routes. The question is therefore not simply whether a molecule “works,” but how its structural, chemical, and electronic functions are expressed within a specific PeLED architecture.

*Expanding and matching the materials space* On the interfacial side, a broader range of molecular systems remains worth exploring, including new anchoring groups, linker geometries, multidentate motifs, and terminal functionalities with more controllable dipolar, coordinative, and wetting properties. On the perovskite side, the compatibility between interface molecules and different emissive materials still requires closer attention, especially in quasi-2D, mixed-halide, nanocrystalline, and deep-blue systems, where small changes near the interface can readily alter phase distribution, carrier confinement, and non-radiative loss. In this sense, future progress is likely to depend not only on developing better interfacial molecules or better perovskites separately, but also on understanding how the two are matched.

*Revisiting device architectures and optical management* As material and interface quality improve, device design itself becomes more consequential. Simpler stacks, fewer functional layers, and more efficient optical outcoupling structures may all become increasingly attractive, provided that charge balance and interfacial robustness can still be maintained. In parallel, the relation between interfacial molecular regulation and device architecture deserves more attention, since the same molecule may behave differently in conventional multilayer devices, reduced-layer structures, or architectures designed for improved light extraction. For PeLEDs, interface design and structural design are therefore unlikely to remain independent problems.

*Stability under electrical operation and coupled degradation processes* This issue still extends beyond the chemical durability of the interfacial molecule alone. In operating devices, degradation often involves coupled processes, including local heating, charge accumulation, ion redistribution, interfacial reaction, and spectral instability. More stable PeLEDs will likely require materials and interfaces that remain compatible not only at rest, but under sustained electrical operation and high brightness. For SAM-based strategies in particular, future progress will also depend on overcoming limitations related to substrate-dependent assembly, incomplete or non-uniform coverage, interfacial wetting mismatch, and the long-term robustness of molecular packing and anchoring under electrical and thermal stress. This points to the need for better bias-stable interfacial molecules, more intrinsically robust emissive materials, and device structures that reduce electrical and thermal stress at the working interfaces.

*Operando and in situ characterization under electrical bias* A further priority is to develop characterization strategies that directly probe working PeLEDs under realistic electrical operation. Although PLQY, TRPL, and density-dependent PL measurements are indispensable for evaluating the intrinsic photophysical properties of emissive materials, they cannot fully reproduce the spatially asymmetric injection, interfacial carrier accumulation, electric-field effects, Joule heating, and coupled degradation processes present in operating devices. Future progress will therefore benefit from broader use of operando and in situ techniques, including transient electroluminescence, operando ultrafast spectroscopy, thermal imaging under bias, operando GIWAXS, and related interface-sensitive probes. Such methods can help establish more direct links between molecular interface design, recombination-zone evolution, interfacial chemical change, and long-term device failure. In this sense, deeper integration of operando characterization with materials and interface design will be essential for developing a more mechanistic understanding of efficient and stable PeLEDs.

Beyond further refinement of individual SAM systems, future progress may also depend on interfacial strategies that are not limited to conventional monolayer design. Multifunctional molecular layers, polymeric charge-injection or interfacial layers, and hybrid molecular architectures may offer advantages in coverage, interfacial tolerance, and operational robustness that are difficult to achieve with SAMs alone. A further limitation is that molecular selection is still dominated largely by empirical screening. Data-driven approaches may therefore become increasingly important, particularly if high-throughput experiments and more standardized interfacial descriptors can be established. In principle, such methods could help reveal less obvious correlations between molecular structure, interfacial energetics, film formation, and device response, thereby improving the efficiency of molecular discovery. Their practical value, however, will depend on the accumulation of sufficiently large and internally comparable experimental datasets.

Overall, the future development of PeLEDs may depend less on optimizing any single material parameter than on how molecular design, perovskite composition, and device structure are integrated. Interfacial molecules will remain important in this process, but their role is likely to be most meaningful when considered together with the emissive material above them and the device architecture around them. From that perspective, interfacial molecular regulation is not only a route to incremental performance improvement, but also part of a broader framework for constructing more efficient, more stable, and more structurally adaptable PeLEDs.
